# New Middle Pleistocene Hominin Dental Remains From Velika Balanica, Serbia

**DOI:** 10.1002/ajpa.70133

**Published:** 2025-10-06

**Authors:** Predrag Radović, Joshua Lindal, Petar Milovanović, Dušan Mihailović, Mirjana Roksandic

**Affiliations:** ^1^ Department of Archaeology, Faculty of Philosophy University of Belgrade Belgrade Serbia; ^2^ Department of Human Origins Max Planck Institute for Evolutionary Anthropology Leipzig Germany; ^3^ National Museum Kraljevo Kraljevo Serbia; ^4^ Department of Anthropology University of Winnipeg Winnipeg Manitoba Canada; ^5^ Department of Anthropology University of Manitoba Winnipeg Manitoba Canada; ^6^ Center of Bone Biology, Institute of Anatomy, Faculty of Medicine University of Belgrade Belgrade Serbia

**Keywords:** dental morphology, Middle Pleistocene, Neanderthals, Serbia, teeth

## Abstract

**Objective:**

The cave site of Velika Balanica in Sićevo Gorge, Serbia, has previously yielded early Neanderthal dental remains from Layer 3a, dated by thermoluminescence to 285 ± 34 and 295 ± 74 ka. We describe and compare four additional dental specimens recovered from the Middle Pleistocene Layers 3a and 3b of the cave: a right I^1^ (BH‐8), a right I_2_ (BH‐7), an incisor crown fragment (BH‐16), and a left M_3_ (BH‐15).

**Material and Methods:**

The fossil teeth were scanned using micro‐computed tomography (μCT), and the resulting digital models were used to record dimensions and assess internal morphology, including the enamel–dentine junction (EDJ). Morphological traits were analyzed on both outer and internal surfaces. Dental measurements were compared to those of relevant hominin samples.

**Results:**

BH‐8 and BH‐7 show large, robust crowns and roots, consistent with Pleistocene Eurasian hominins. Linear enamel hypoplasia is present in BH‐8 and BH‐16. BH‐15 displays a continuous middle trigonid crest—a trait considered diagnostic of the Neanderthal lineage. Notably, BH‐15 also presents an extreme case of taurodontism, as well as a severe antemortem tooth fracture accompanied by multiple pulp stones, which are rarely recorded in the hominin fossil record.

**Discussion:**

These findings align with earlier results, reinforcing evidence for early Neanderthal presence at Velika Balanica around 300 ka. While Neanderthals may have been present in the region earlier, this represents the earliest dated evidence of their spread into the Balkans.

## 1 Introduction

**FIGURE 1:**
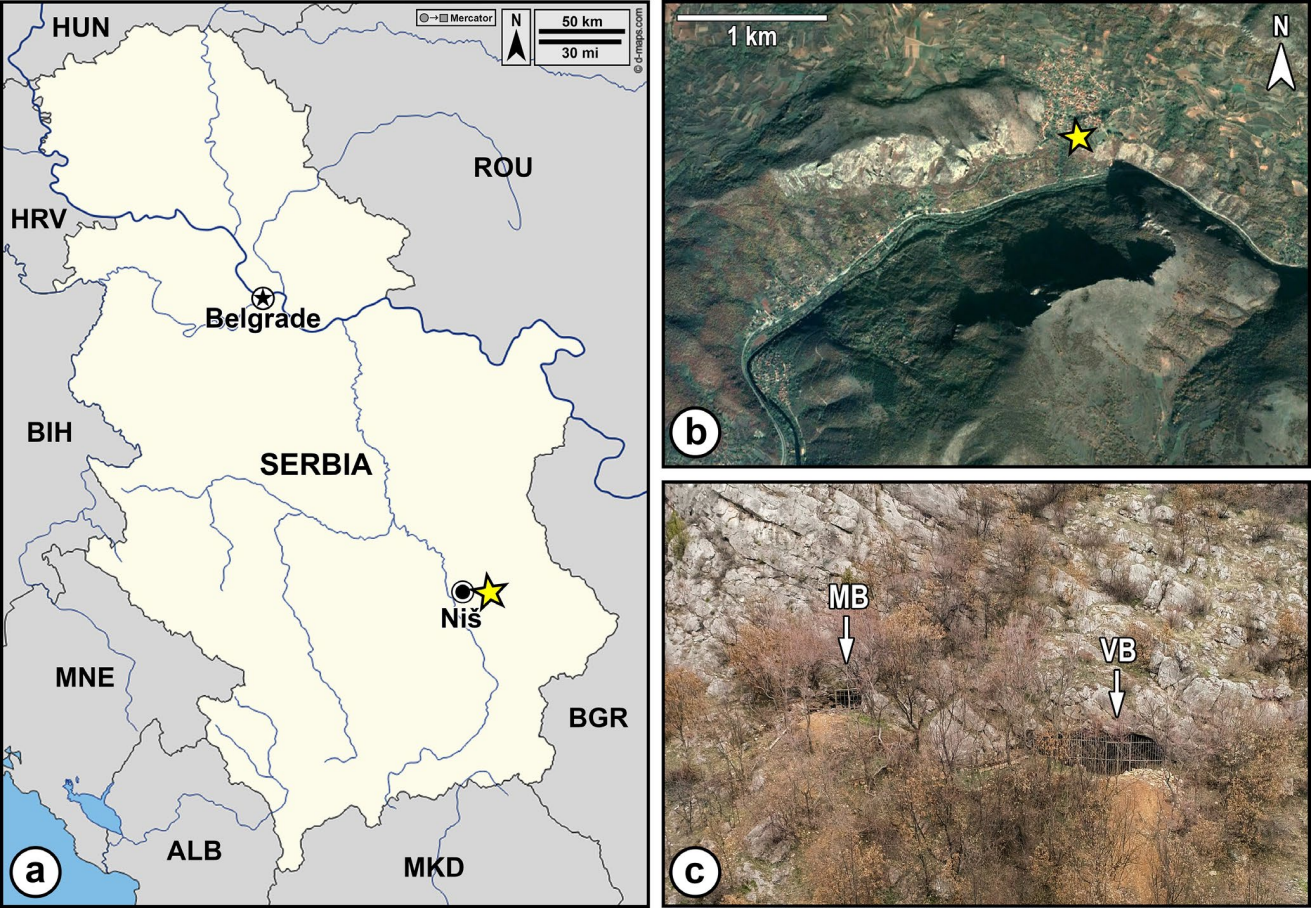
Geographic location of the Balanica Cave Complex in Serbia (a), its position within the Sićevo Gorge (b) marked with yellow stars, and views of the entrances to the Mala Balanica (MB) and Velika Balanica (VB) caves (c). Neighboring countries are abbreviated according to ISO 3166‐1 alpha‐3 codes. The original map was downloaded on February 12, 2025, from https://d‐maps.com/carte.php?num_car=27556&lang=en. The satellite image utilized for this depiction was sourced from Google Earth Pro 7.3.6 (https://www.google.com/earth/versions/) on March 10, 2024.

Situated within the Sićevo Gorge near Niš in southern Serbia (43°20.211'N, 22°05.115′E), the Balanica Cave Complex comprises two extensively studied caves: Mala Balanica and Velika Balanica (Figure 1). Both caves have provided intriguing evidence for Middle Pleistocene (Chibanian) hominins in the area. Older than 392–525 ka (Rink et al. 2013), the BH‐1 hemi‐mandible from Mala Balanica lacks a Neanderthal morphological signal (Roksandic et al. 2011; Skinner et al. 2016) and exhibits primitive features similar to those of Middle Pleistocene specimens from Southwest Asia (Roksandic et al. 2018). In Velika Balanica, several layers with Middle Paleolithic artifacts (Layers 2a–2c, 3a–3c) have been excavated over an area of approximately 30 m^2^. Primarily composed of compact silt and containing evidence of intense burning, Layer 3 has yielded the majority of artifacts and faunal remains (Mihailović, Kuhn, et al. 2022), as well as a small collection of hominin fossils described by Roksandic et al. (2022). The latter, deriving from the upper part of Layer 3 (i.e., Layer 3a)—which was dated by thermoluminescence (TL) of burnt lithic artifacts to 285 ± 34 and 295 ± 74 ka (Mihailović, Kuhn, et al. 2022)—represent at least two individuals and comprise a heavily worn left M^3^ (BH‐2), a heavily worn and fragmentary right deciduous P^4^ (BH‐3) that has been virtually re‐fitted with a poorly preserved maxillary fragment containing an unworn M^1^ in the alveolus (BH‐4), and a heavily worn left I^1^ (BH‐5) specimen (Roksandic et al. 2022). The archaeological finds from Layer 3 have been attributed to the early Quina complex and linked to contemporaneous Yabrudian sites in the Levant, including Yabrud, Qesem, Tabun, Misliya, and El Kowm (Le Tensorer 2005; Shimelmitz et al. 2014; Zaidner and Weinstein‐Evron 2016; Barkai et al. 2017). Unlike the Levantine sites, where hominin remains from this period have been confirmed but not yet definitively taxonomically identified, the hominin remains from Layer 3 of Velika Balanica have been identified as early Neanderthals, morphologically similar to the approximately 430,000‐year‐old Sima de los Huesos hominins (Roksandic et al. 2022).

**FIGURE 2:**
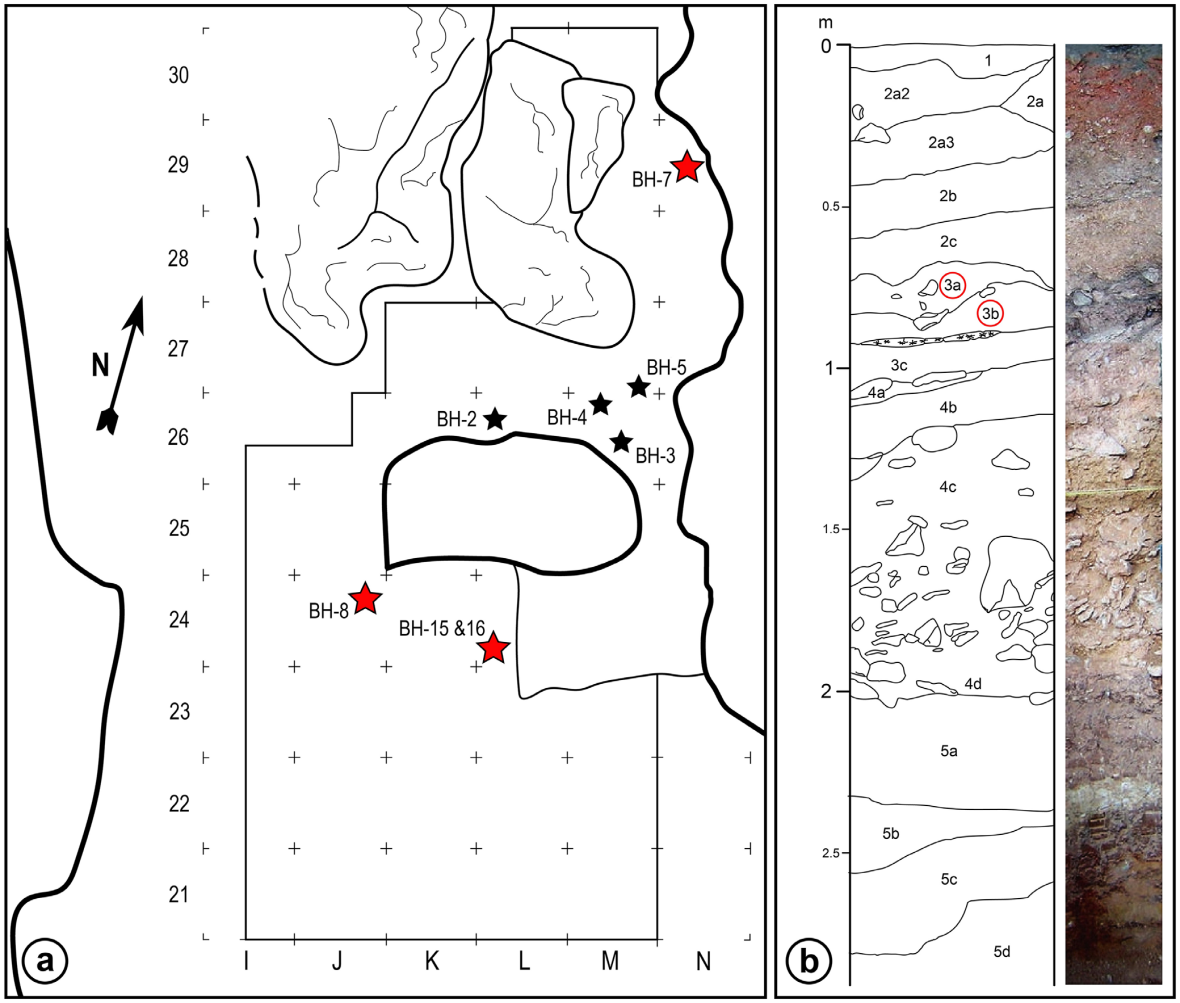
Plan of the excavated area in the Velika Balanica cave showing the square grid and approximate positions of hominin fossils (a), and the stratigraphy of the north profile within square M23 (b). Black stars indicate previously published hominin remains (Roksandic et al. 2022), while those described in this study are marked with red stars. Labels of hominin‐bearing layers are encircled in red. The stratigraphic column was adapted from Mihailović and Bogićević (Mihailović and Bogićević 2016, Figure 9.5).

Here, we describe and analyze additional hominin dental remains from Layer 3 of Velika Balanica, identified in the recent laboratory and field research and originating from the same area along the eastern wall of the cave as the previously published specimens: an isolated hominin incisor, BH‐7, excavated in the 2020 field season from Layer 3b of Velika Balanica; an isolated incisor, BH‐8, unearthed in 2012 from Layer 3b; an isolated molar, BH‐15, and an incisor crown fragment, BH‐16, both excavated in 2011 from Layer 3a. Table 1 and Figure 2 provide field data on all hominin fossil specimens found at Velika Balanica. The similar lithological composition and shared characteristics of the finds across all three sublevels of Layer 3 (i.e., 3a, 3b, and 3c; Mihailović, Kuhn, et al. 2022) suggest that the specimens from Layer 3b (BH‐7 and BH‐8) are unlikely to be significantly older than those from Layer 3a. Therefore, we test the hypothesis that the new dental remains from Velika Balanica have a similar taxonomic affinity to that of the previously published specimens.

**TABLE 1:** Field data on hominin remains recovered from Velika Balanica so far.

Specimen	Element	Geological layer	Square, quadrant	Provenience, field inventory number	Year excavated
BH‐2	Left M^3^	3a	L26, b	Sieve	2017
BH‐3	Right DP^4^	3a	M26, b	Moved	2017
BH‐4	Right maxillary fragment with M^1^	3a	M26, b	In situ, 1	2017
BH‐5	Left I^1^	3a	M27, a	In situ, 11	2017
**BH‐7**	**Right I**_**2**_	3b	N29, b	In situ, 60	2020
**BH‐8**	**Right I** ^**1**^	3b	J24, c	In situ, 4	2012
**BH‐15**	**Left M**_**3**_	3a	L24, a	Sieve	2011
**BH‐16**	**Incisor crown fragment**	3a	L24, a	Sieve	2011

*Note:* Newly described specimens are highlighted in bold.

## 2 Methods

The fossil teeth were scanned by micro‐computed tomography (μCT) at the Center of Bone Biology, Institute of Anatomy, Faculty of Medicine, University of Belgrade (Serbia) using a SkyScan 1172 microtomograph (Bruker microCT, Kontich, Belgium) with a source voltage of 89 kV, a source current of 112 μA, exposure time of 1200 ms, and an aluminum–copper filter (0.5 mm Al + 0.04 mm Cu), at a resolution of 9.94 μm, frame averaging of 3, and a 0.45° rotation step. The raw data were reconstructed using NRecon software v.1.6.9.8 (Bruker microCT), with a smoothing of 3, beam hardening correction of 20%, and appropriate ring artifact and post‐alignment corrections. The final volumes for the three specimens were reconstructed with an isotropic voxel size of 9.94 μm.

Mesiodistal (MD), labiolingual (LaL, for anterior dentition), and buccolingual (BL, for postcanine dentition) crown and cervical diameters of the fossil teeth were measured by one of the authors (J.L.) on isosurface renderings of individual teeth using the measurement tool in Amira 3D (v.2021.1, FEI SAS, Thermo Fisher Scientific Inc.). Diameters were recorded at the broadest points in the crown and cervix to the nearest 0.1 mm. Incisor root length (RL) was recorded from the apex to the center of the root canal at the cervical plane, defined as the plane that intersects the apicalmost points on the cementum–enamel junction (CEJ) on the buccal and lingual aspects, following Le Cabec et al. (2013). General dental terminology follows Scott et al. (2018). Tooth wear was assessed according to Molnar's system (Molnar 1971). Dental morphological traits were scored according to the modified ASUDAS system proposed by Martinón‐Torres et al. (2012). Segmentation of enamel and dentin for all teeth was conducted in Amira 3D on the median‐filtered image stacks using a combination of automatic thresholding and manual corrections.

We compared the crown and root dimensions of BH‐8 and BH‐7 (i.e., MD and LaL crown diameters, root length) as well as the BL crown diameter of BH‐15 to samples of early Neanderthals (i.e., Sima de los Huesos), late Neanderthals, and modern humans—Middle Paleolithic *Homo sapiens* (i.e., Qafzeh) and Upper Paleolithic *H. sapiens* for crown diameters, and Upper Paleolithic to Epipaleolithic *H. sapiens* and recent *H. sapiens* samples for root lengths—using bivariate plots and adjusted *Z*‐scores (Maureille et al. 2001; Scolan et al. 2012). The use of adjusted *Z*‐scores is particularly well suited for fossil data, as this statistical method enables the comparison of unbalanced and small samples by applying the inverse Student's t‐distribution following the formula: [(*x* − *m*)/(s*sqrt(1 + 1/*n*))]/(Student.t.inverse(0.05; *n* − 1)), where *x* is the value of the variable being tested (e.g., MD crown diameter of BH‐8), *m* is the mean of the same variable for a comparative sample (e.g., MD crown diameter for late Neanderthals), *n* is the size of the comparative sample, and *s* is the standard deviation of the comparative sample. In these adjusted *Z*‐scores, the interval from −1.0 to +1.0 encompasses 95% of the variation within the reference sample (also see Zanolli 2013). For crown diameters, comparative specimens and their respective measurements are listed in Tables S1–S3. Root length data are primarily taken from Le Cabec et al. (2013, Table 4b)—with the original group names “Neanderthals,” “Early modern humans,” “Upper Paleolithic and Epipaleolithic humans,” and “Recent modern humans” here renamed as “late Neanderthals,” “Upper Paleolithic and Epipaleolithic *Homo sapiens* ,” and “recent *Homo sapiens* ,” respectively—except for early Neanderthal lower lateral incisors from Sima de los Huesos, which are sourced from Lockey et al. (2023, Table 4). A bivariate plot was created using PAST 4.17 (Hammer et al. 2001).

## 3 Results

### 3.1 Descriptions

**FIGURE 3:**
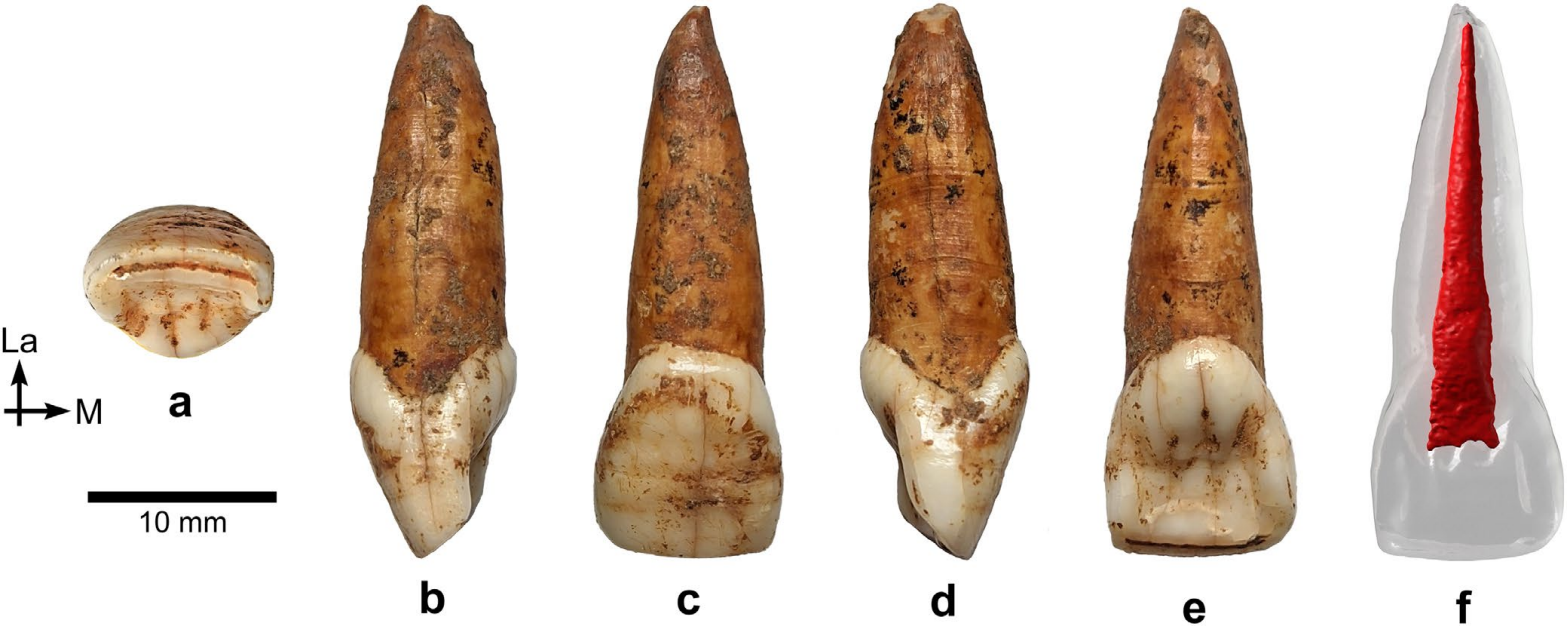
Right I^1^ BH‐8 in occlusal (a), mesial (b), labial (c), distal (d), and lingual (e) views, based on photographs of the original specimen, and as a semitransparent μCT‐based 3D model showing the red‐colored pulp cavity in lingual view (f). Abbreviations: La = labial; M = mesial.

**FIGURE 4:**
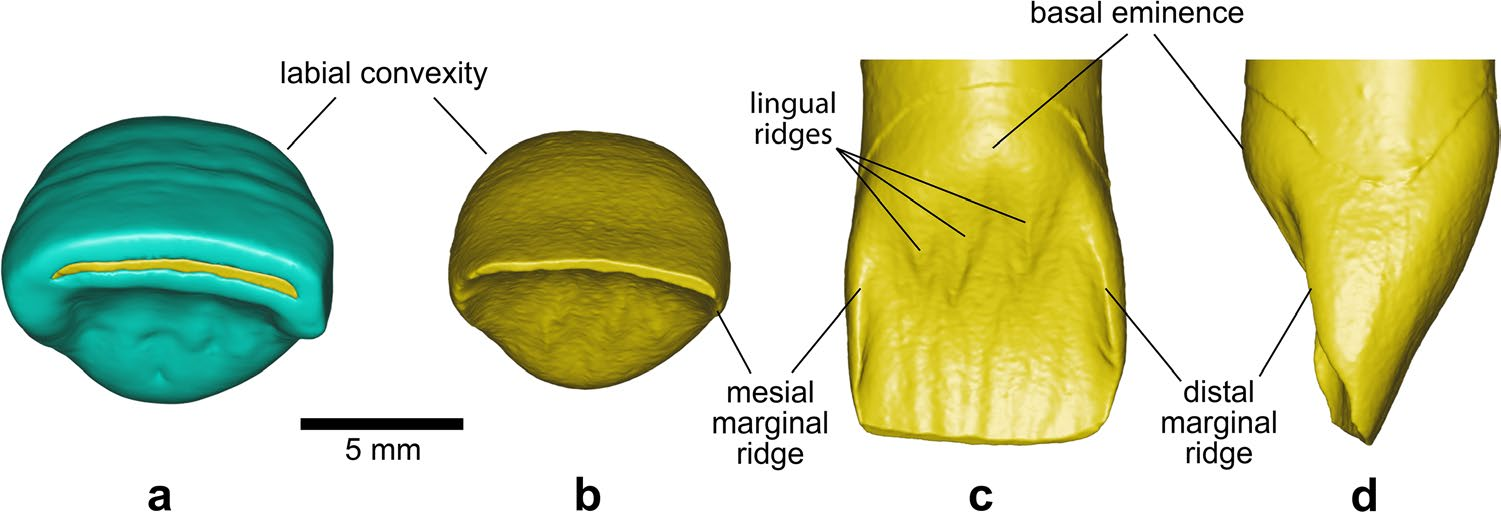
Virtual renderings of the BH‐8 incisor crown based on μCT data, illustrating the dental morphological traits discussed in the text. Occlusal views of the outer enamel surface (OES) (a) and enamel–dentine junction (EDJ) (b); lingual (c), and distal (d) views of the EDJ. In the occlusal views, the labial side is oriented upward. Enamel is shown in blue and dentine in yellow.

*BH‐8* is a permanent upper right central incisor (RI^1^) in an excellent state of preservation, with minimal damage to the root apex (Figure 3). This incisor, as well as the three other specimens described in this paper, exhibits black staining, likely caused by manganese oxide precipitation—a common occurrence in cave settings (López‐González et al. 2006). The complete crown shows only slight wear, exposing a thin linear patch of dentine (Stage 3; Molnar 1971). Several horizontal furrow‐type enamel defects can be seen with the naked eye on the labial surface of the BH‐8 crown (Figure 3c), representing clear evidence of linear enamel hypoplasia (LEH) in this individual (Hillson and Bond 1997). Viewed from the occlusal aspect (Figures 3a and 4a,b), the crown shows pronounced labial convexity (Grade 4; Martinón‐Torres et al. 2012). The mesial and distal marginal ridges are clearly visible and well defined on the lingual surface of the crown—both on the outer enamel surface (OES) and the enamel–dentine junction (EDJ), as illustrated in Figures 3e and 4c—forming a moderate shovel shape (Grade 3; Martinón‐Torres et al. 2012). Furthermore, the lingual cingular region shows *tuberculum dentale*, expressed as a clear basal eminence with three distinct ridges (Grade 3; Martinón‐Torres et al. 2012) (Figures 3d,e and 4c,d). Dental measurements for this specimen are presented in Table 2, while Table 3 shows the expressions of dental morphological traits.

**TABLE 2:** Crown and cervical diameters and root lengths (in mm) of the Velika Balanica teeth described in this study.

Specimen	Tooth category	MD crown diameter	LaL/BL crown diameter	MD cervical diameter	LaL/BL cervical diameter	Root length
BH‐8	Right I^1^	10.2	8.8	7.9	7.8	17.9
BH‐7	Right I_2_	—	8.1	5.3	7.8	17.8
BH‐15	Left M_3_	—	10.2	9.6	9.2	—

Abbreviations: BL = buccolingual; LaL = labiolingual; MD = mesiodistal.

**TABLE 3:** Expression of the main dental morphological traits in BH‐8, BH‐7, BH‐16, and BH‐15, according to the modified ASUDAS system proposed by Martinón‐Torres et al. (2012).

Specimen	Dental trait	Grade (definition)
BH‐8	Labial convexity	4 (Labial surface exhibits pronounced convexity.)
	Shovel shape	3 (Moderate shovel shape. The marginal ridges are more pronounced and there is a tendency for ridge convergence.)
	*Tuberculum dentale*	3 (Strong ridging.)
BH‐7	Labial convexity	4 (Labial surface exhibits pronounced convexity.)
	Shovel shape	1 (Faint shovel shape. The mesial and distal aspects of the lingual surface can be seen and palpated.)
	*Tuberculum dentale*	1 (Faint ridging.)
BH‐16	Labial convexity	Possibly 4 (Labial surface exhibits pronounced convexity.)
BH‐15	Middle trigonid crest	2 (There is a continuous bridge‐like crest connecting the mesial aspects of the protoconid and the metaconid.)
	Distal trigonid crest	1 (The crest is weak or interrupted by the central groove; it may be identified by the expression of a short fissure in the protoconid parallel to the buccolingual fissure.)
	Deflecting wrinkle	0 (The metaconid crest is straight.)*
	Hypoconulid size	3 (The hypoconulid is medium‐sized.)
	C6 (entoconulid or *tuberculum sextum*)	0 (The C6 is absent.)
	C7 (metaconulid or *tuberculum intermedium*)	0 (The C7 is absent.)
	Groove pattern	2 [A groove pattern different from the Y‐pattern is present (“X”: C1 and C4 are in contact, or “+”: four main cusps are in contact.)]
	Protostylid	0 (No expression of any sort.)

*Note:* Features estimated using both the OES and EDJ surfaces.

* In their original definition of the deflecting wrinkle, Martinón‐Torres et al. (2012, Table 4) refer to the “essential crest of the paraconid” and “paraconid crest,” which is evidently an error, as the correct terms should be “essential crest of the metaconid” and “metaconid crest.”

**FIGURE 5:**
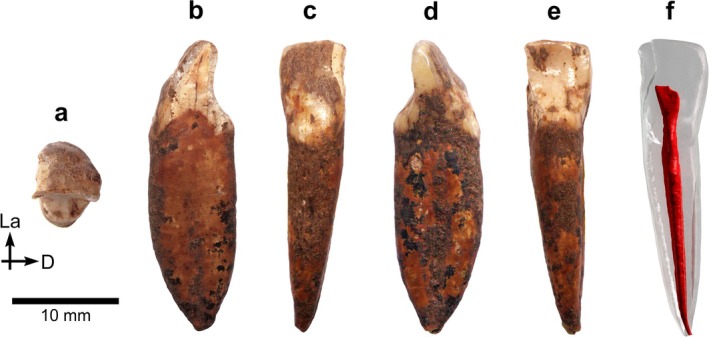
Right I_2_ BH‐7 in occlusal (a), mesial (b), labial (c), distal (d), and lingual (e) views, based on photographs of the original specimen, and as a semitransparent μCT‐based 3D model showing the red‐colored pulp cavity in lingual view (f). Abbreviations: D = distal; La = labial.

**FIGURE 6:**
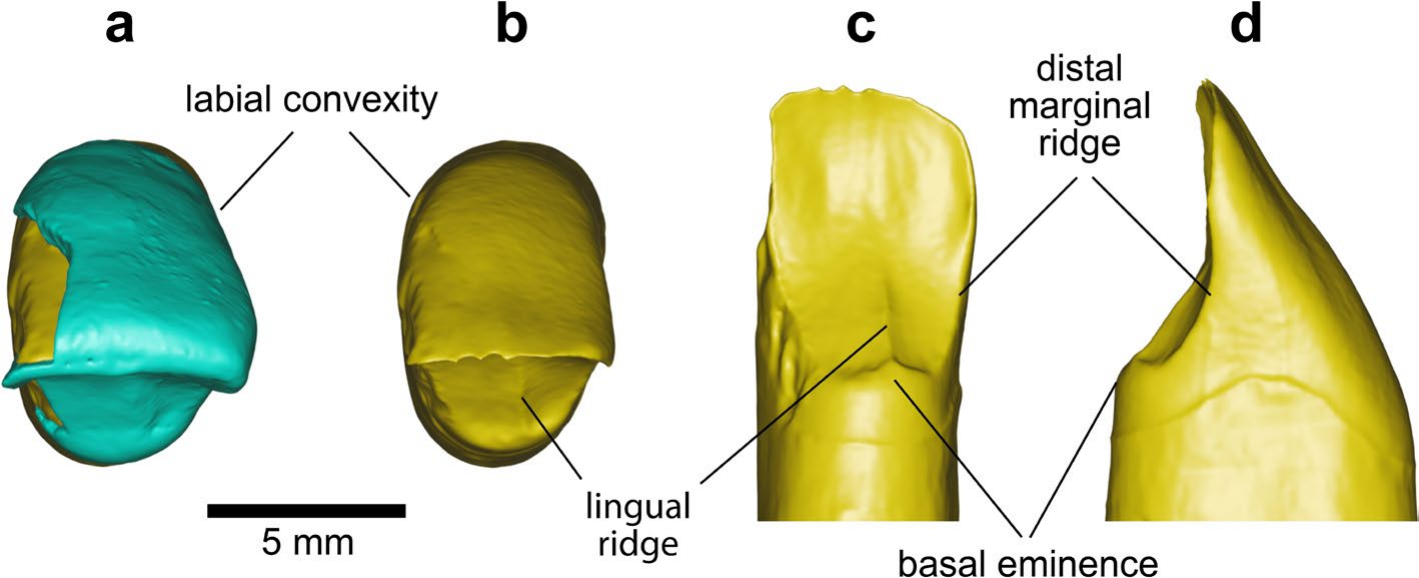
Virtual renderings of the BH‐7 incisor crown based on μCT data, illustrating the dental morphological traits discussed in the text. Occlusal views of the OES (a) and EDJ (b); lingual (c) and distal (d) views of the EDJ. In the occlusal views, the labial side is oriented upward. Enamel is shown in blue and dentine in yellow.

*BH‐7* is a permanent lower right lateral incisor (RI_2_) preserving a complete root and a crown missing the mesialmost portion of the enamel due to postmortem damage (Figure 5). The specimen exhibits only the beginnings of wear, with a minimal wear facet present (Stage 2 following Molnar 1971). In occlusal view (Figures 5a and 6a,b), the crown displays a pronounced labial convexity (Grade 4 following Martinón‐Torres et al. 2012). A very weak distal marginal ridge (suggestive of Grade 1 shovel shape; Martinón‐Torres et al. 2012), along with a clear basal eminence and faint ridging in the region of the cingulum (Grade 1 *tuberculum dentale*; Martinón‐Torres et al. 2012), can be observed both in the OES and EDJ (Figure 6). The root is long and displays strong mesiodistal compression. There is a notable distal tilt of the crown relative to its mid‐root axis (see Scheid and Weiss 2016: 66). In mesial and distal views, the root is wide, with broad, shallow longitudinal grooves along the mesial and distal face (with the distal groove being slightly deeper and broader), delimiting the labial and lingual root elements. Dental measurements are provided in Table 2, while the expressions of dental traits are presented in Table 3.

**FIGURE 7:**
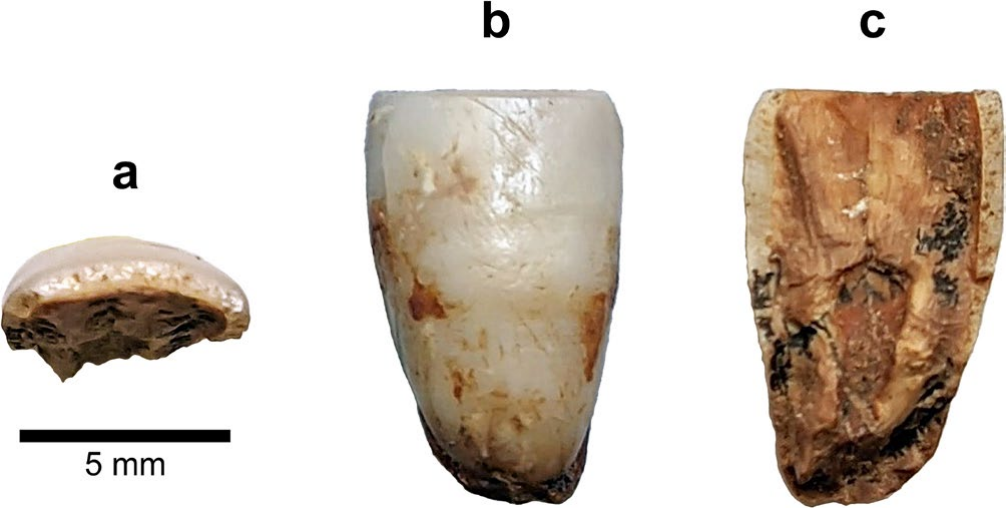
Incisor crown fragment BH‐16 in occlusal (a), labial (b), and lingual (c) views, based on photographs of the original specimen.

**FIGURE 8:**
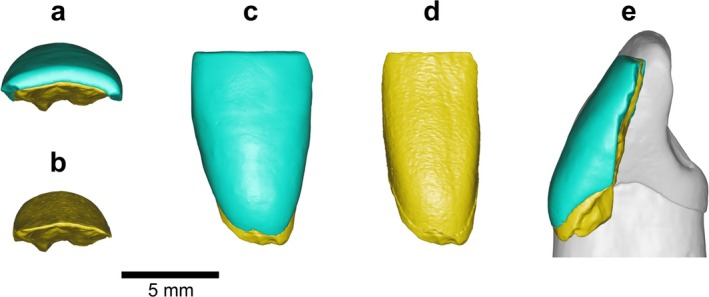
Virtual renderings of the BH‐16 incisor fragment based on μCT data. Occlusal views of the OES (a) and EDJ (b); labial views of the OES (c) and EDJ (d); a mesial/distal view superimposed on the mirrored distal view of BH‐7 in grayscale (e). In occlusal views, the labial side is oriented upward. Enamel is shown in blue, and dentine in yellow.

*BH‐16* is an incisor crown fragment with nearly the entire labial surface preserved and a visible CEJ (Figures 7 and 8). The fragmentation is not recent, as evidenced by the manganese coating on the exposed internal crown (Figure 7c). The incisal edge exhibits signs of wear, while the labial surface displays distinct LEHs. The breakage reveals the internal structure in the lingual view—the pulp chamber is mesiodistally broad and follows the external crown contour (Figure 7c). The fragment is 6.2 mm wide mesiodistally and about 10 mm high. Although the specimen is too fragmented to allow a conclusive determination of its position, the mesiodistally narrow crown suggests that BH‐16 may represent a lower incisor. Furthermore, BH‐16 closely resembles BH‐7 in morphology and size (see Figure 8e), but likely originates from the opposite side of the jaw, suggesting it may be a left I_2_. Additionally, BH‐16 is more worn than BH‐7, with dentine exposure already apparent. Labial convexity was possibly pronounced (Grade 4; Martinón‐Torres et al. 2012) in this specimen (see Figure 8a,b); however, fragmentation precludes a reliable assessment.

**FIGURE 9:**
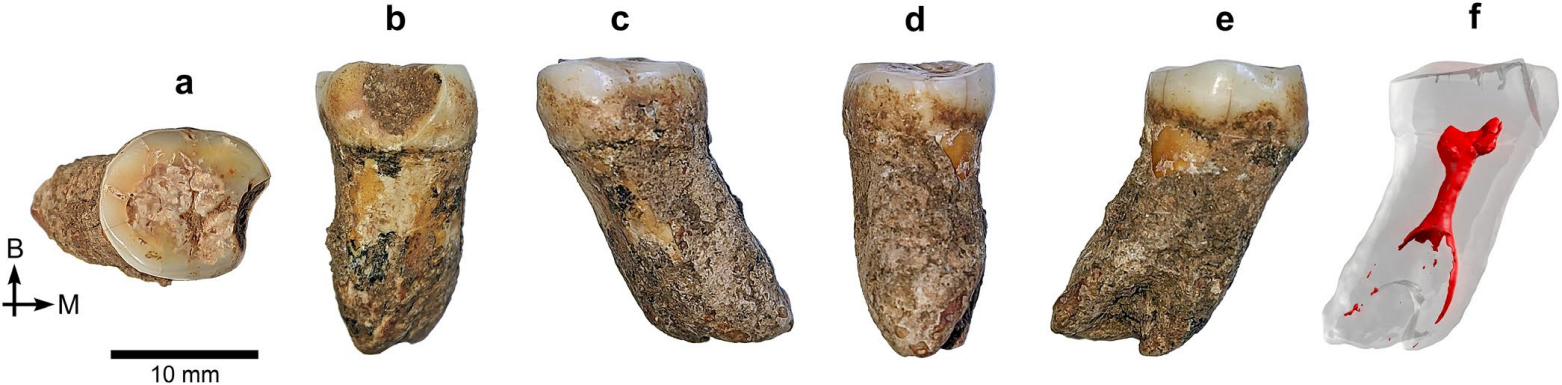
Left M_3_ BH‐15 in occlusal (a), mesial (b), buccal (c), distal (d), and lingual (e) views based on photographs of the original specimen, and as a semitransparent μCT‐based 3D model showing the red‐colored pulp cavity in lingual view (f). Abbreviations: L = lingual; M = mesial.

**FIGURE 10:**
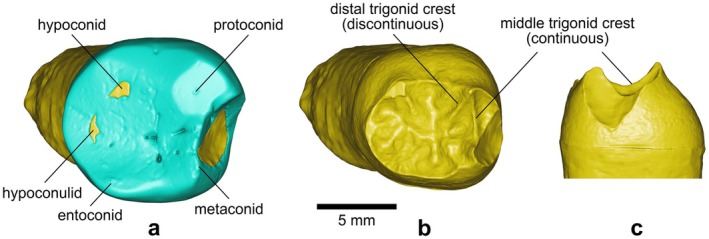
Virtual renderings of BH‐15 based on μCT data, illustrating the dental morphological traits discussed in the text. Occlusal views of the OES (a) and EDJ (b); mesial view of the EDJ (c). In the occlusal views, the buccal side is oriented upward. Enamel is rendered in blue and dentine in yellow.

*BH‐15* is a lower left third molar (LM_3_) preserving complete roots and a damaged crown, with a significant portion of the mesial (mostly metaconid) enamel and dentine missing (Figure 9). In the mesial and distal views (Figure 9b,d), the specimen shows a slight lingual tilt of its crown relative to its midroot axis line—a characteristic of posterior lower teeth (Scheid and Weiss 2016: 147), which, together with the rounded rectangular occlusal outline of the crown, the absence of a distal interproximal contact facet, the strong distal angulation of the root trunk, and the root fusion (with buccal root furcation set very low), is consistent with an *M*_3_. The roots of BH‐15 exhibit a notable degree of taurodontism. Various methods exist to measure/score taurodontism: Shifman and Chanannel's (1978) method yields a Taurodont Index (TI) of 45.7, while Seow and Lai's (1989) method yields a crown‐body/root ratio of 3.0; both represent the most extreme category of expression, that is, “hypertaurodontism” *sensu* Shaw (1928). Additionally, the root branches are “fused” (developmentally contiguous) across their buccal side, creating a distinctive “C‐shaped” morphology in cross‐section (Cooke and Cox 1979). Despite enamel damage and wear (Stage 3; Molnar 1971) obscuring some morphological details on the OES of BH‐15, the central fossa, buccal and lingual grooves, and the deeper portions of the distal central groove remain visible (Figures 9a and 10a). With four of the five main cusps (protoconid, metaconid, hypoconid, and entoconid) in contact at the central groove, the BH‐15 crown displays the cruciform or “+” groove pattern (Grade 2; Martinón‐Torres et al. 2012). At the EDJ, the molar shows dentine horns corresponding to the five main cusps (with the hypoconid and hypoconulid horns noticeably worn), a low but continuous middle trigonid crest (MdTC), and a discontinuous distal trigonid crest (DTC) interrupted by the central groove (see Figure 10). The morphology of the preserved EDJ region mesial to the MdTC suggests the presence of an anterior fovea; however, crown damage prevents accurate characterization of this feature (Figure 10b). The dental measurements for BH‐15 are presented in Table 2, while Table 3 shows the expressions of dental morphological traits.

**FIGURE 11:**
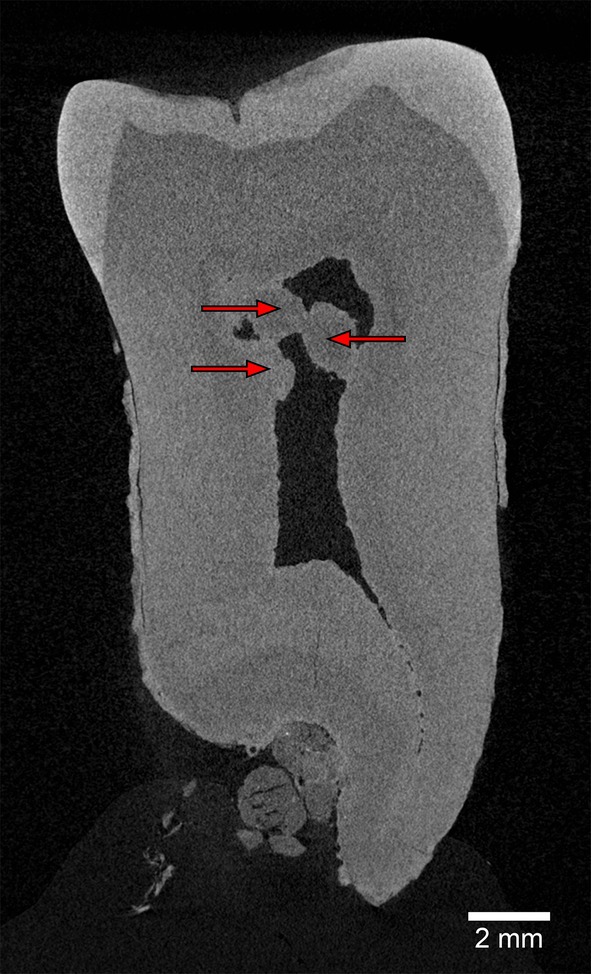
Buccolingual section of the BH‐15 molar (μCT slice) showing multiple calcified bodies within the pulp chamber, indicated by red arrows.

As shown in Figure 9b, the fracture in the mesial part of the BH‐15 crown features smooth edges and a polished surface, an accumulation of calculus, and a coloration distinct from the lighter hues of freshly broken enamel and dentine (e.g., as seen in the postmortem damage to the BH‐7 crown; Figures 5 and 6a–c). These characteristics strongly indicate that the fracture occurred during the individual's lifetime (see Milner and Larsen 1991; Scott and Winn 2011). The damage to the crown is severe, affecting both the enamel and dentin, and aligns with the Grade 3 chipping described by Belcastro et al. (2018: 98), defined as a “crack bigger than 1 mm involving enamel and dentin or a large, very irregular fracture that could destroy the tooth.” As Figure 11 shows, multiple discrete calcified bodies are deposited in the pulp chamber of the BH‐15 molar. Referred to as pulp stones, denticles, endoliths, or pulpoliths (Palatyńska‐Ulatowska et al. 2019), these calcifications are composed of dentine, lined by odontoblasts, and may be found freely within the pulp tissue, attached to, or embedded in the dentine (Johnson and Bevelander 1956; Goga et al. 2008). The pulp stones in BH‐15 appear as multiple oblong masses that merge together, occupying a large proportion of the coronal pulp chamber, adhering to the buccal and lingual walls.

### 3.2 Metric Comparisons

**FIGURE 12:**
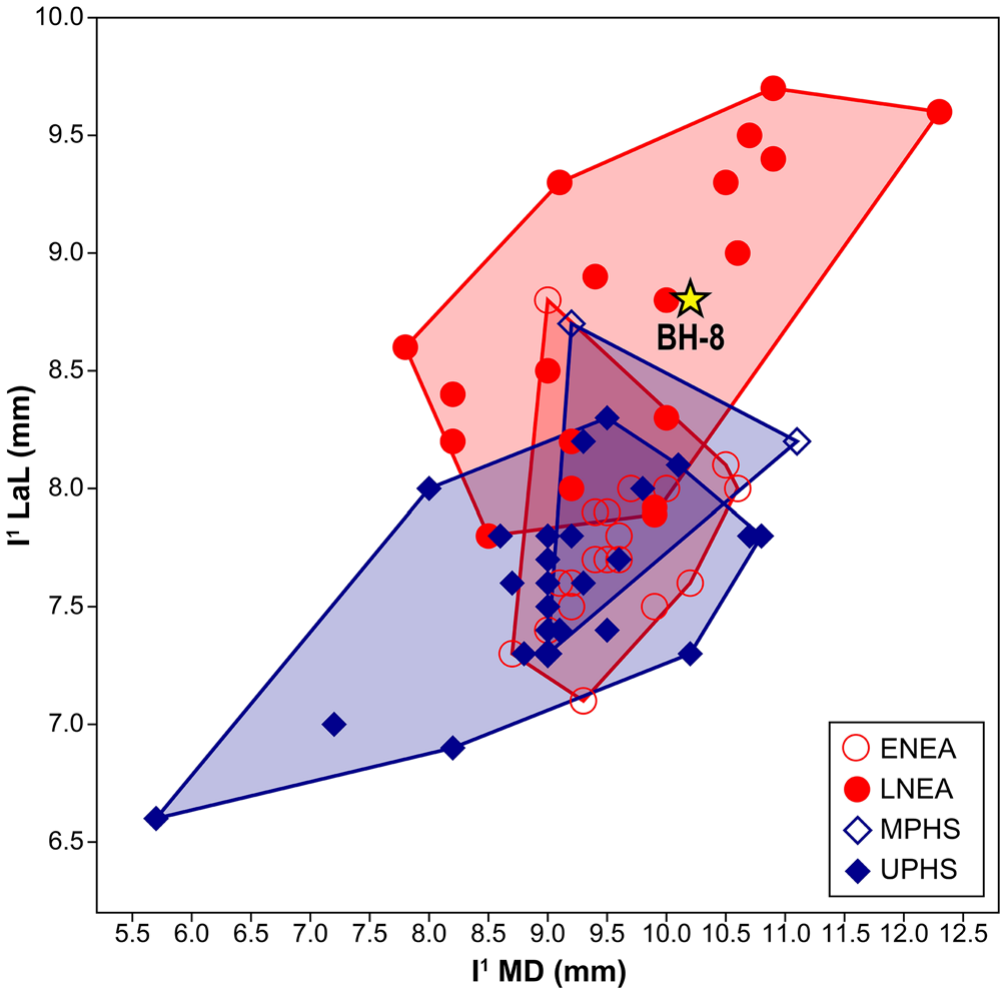
Bivariate plot of mesiodistal (MD) and labiolingual (LaL) crown diameters (in mm) for the BH‐8 upper central incisor (yellow star), compared to Neanderthal and modern human I^1^ samples. Abbreviations: ENEA = early Neanderthals (i.e., Sima de los Huesos sample); LNEA = late Neanderthals; MPHS = Middle Paleolithic *Homo sapiens* (i.e., Qafzeh sample); UPHS = Upper Paleolithic *H. sapiens* . For the list of comparative specimens and their respective measurements, see Table S1.

**FIGURE 13:**
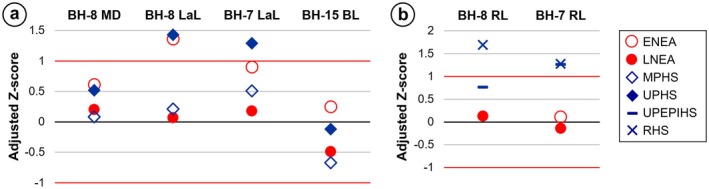
Adjusted *Z*‐scores of the crown (a) and root (b) diameters of the new teeth from Velika Balanica (RI^1^ BH‐8, RI_2_ BH‐7, and LM_3_ BH‐15) compared to the variation of Neanderthal and modern human samples. The black line at zero represents the mean, while the red lines at +1 and −1 correspond to the estimated 95% limit of the variation of the comparative samples. For crown diameters, the comparative specimens and their respective measurements are listed in Tables S1 **–** S3. For root lengths, the data are sourced from Table 4b in Le Cabec et al. (2013), except for the early Neanderthal I_2_s, which are sourced from Table 4 in Lockey et al. (2023). The figure is based on data presented in Table S4. Abbreviations: BL = buccolingual crown diameter (breadth); ENEA = early Neanderthals (i.e., Sima de los Huesos sample); LaL = labiolingual crown diameter (breadth); LNEA = late Neanderthals; MD = mesiodistal crown diameter (length); MPHS = Middle Paleolithic *Homo sapiens* (i.e., Qafzeh); RHS = recent *H. sapiens* ; RL = root length; UPHS = Upper Paleolithic *H. sapiens* ; UPEPIHS = Upper Paleolithic and Epipaleolithic *H. sapiens* .

The results of the metric comparisons, based on adjusted *Z*‐scores and a bivariate plot, are presented in Figures 12 and 13. Regarding crown measurements, BH‐8 does not show any statistically significant differences from any of the comparative groups in mesiodistal diameter, but it does differ from the Sima de los Huesos and Upper Paleolithic *H. sapiens* samples in labiolingual diameter; however, the values for both diameters in BH‐8 fall close to the mean values of late Neanderthals and Middle Paleolithic *H. sapiens* samples. BH‐7 exhibits a statistically significant difference in labiolingual diameter compared to the Upper Paleolithic *H. sapiens* sample, with the value being closest to the mean of late Neanderthals. The buccolingual breadth of the BH‐15 molar does not differ significantly from that of the comparative groups. The roots of the two incisors are long—BH‐8 shows a statistically significant difference from the recent *H. sapiens* sample, while BH‐7 is significantly different from both Upper Paleolithic and Epipaleolithic and recent *H. sapiens* samples. The BH‐8 root is also longer (*RL* = 17.9 mm) than the previously described upper central incisor from Velika Balanica (BH‐5, *RL* = 16.4 mm; Roksandic et al. 2022).

## 4 Discussion

New fossil specimens from Layer 3 of the Velika Balanica cave are consistent with Neanderthal taxonomic classification and offer further insights into the morphological dental variation of the early Neanderthals in the Balkans. The massive and robust structure of the BH‐8 and BH‐7 incisors aligns with a dental morphological pattern characteristic of Eurasian Pleistocene hominins (Martinón‐Torres et al. 2007; Bermúdez de Castro et al. 2019). BH‐7 and BH‐8 display large crowns, with MD and LaL diameters closest to the mean values of (late) Neanderthals and Middle Paleolithic *H. sapiens* (Figures 12 and 13). These two specimens (and possibly also BH‐16) display pronounced labial convexity (Grade 4; Martinón‐Torres et al. 2012)—an incisor trait typical of members of the Neanderthal lineage (e.g., Bailey 2000, 2006; Bermúdez de Castro et al. 2021a, 2021b; Najafzadeh et al. 2024). A pronounced labial convexity was also observed in BH‐5, remaining clearly visible despite heavy wear (Roksandic et al. 2022). Recorded expressions of shovel shape and *tuberculum dentale* in BH‐8 and BH‐7 (Table 3) are also consistent with Neanderthal variation (see Martinón‐Torres et al. 2012). As demonstrated by Le Cabec et al. (2012, 2013), the roots of late Neanderthal anterior teeth differ from those of recent modern humans in being larger (in terms of root length, surface areas, and root volume variables) and also show different root shapes for each tooth type. The BH‐8 and BH‐7 roots are long and distinct from those of modern human samples, falling very close to the means for Neanderthals (Figure 13). This pattern is also evident in the previously described BH‐5 (Roksandic et al. 2022), where the root length closely matches that of late Neanderthals and differs from the shorter roots of recent *H. sapiens* (Table S4). Hominin incisors from Layer 3 of Velika Balanica display linear enamel hypoplasia (LEH) (Figures 3c and 7b), a disruption of ameloblast function commonly associated with nonspecific stress during the secretory stage of enamel formation (Hillson and Bond 1997; Hillson 2014; Towle and Irish 2020; McGrath et al. 2021). Deeper LEHs have been linked to more severe stress events in humans and other mammals (e.g., Suckling and Thurley 1984; Goodman and Rose 1990; Moggi‐Cecchi et al. 1994; McGrath et al. 2018, 2021). As important markers of systemic physiological stress during childhood, LEHs have been documented in various hominin groups (e.g., Reid and Dean 2000; Guatelli‐Steinberg 2003, 2004; Skinner 2019; Towle and Irish 2019), including Neanderthals (Cunha et al. 2004; Hlusko et al. 2013; Guatelli‐Steinberg et al. 2014). Further microscopic studies of the LEH in Velika Balanica hominin dentition are underway.

The expression of a middle trigonid crest (MdTC) in the EDJ of BH‐15 is potentially taxonomically informative. Trigonid crests refer to the expression of a ridge or crest connecting the mesial cusps (protoconid and metaconid) in hominid lower molars (Scott et al. 2018: 51). These crests vary in terms of presence (absent, interrupted, or continuous), height (low or high), and origin, with the variability at EDJ being greater than that on the OES. They can arise from the mesial, middle (or essential), and distal segments of the mesial cusps or sporadically from the marginal ridge complex (Bailey et al. 2011; Martínez de Pinillos et al. 2014). Depending on whether they connect the mesial/middle or distal segments of mesial cusps, they are termed middle or distal trigonid crests, respectively (abbreviated MdTC and DTC, respectively), and both crest types are observable at the OES (Martínez de Pinillos et al. 2014). In those cases when one of the MdTC ends reaches the mesial marginal ridge, the more proper term would be mesial trigonid crest (MeTC)—but this distinction can only be made at EDJ (Martínez de Pinillos et al. 2014). A continuous MdTC is regarded as a diagnostic trait of the Neanderthal lineage (Bailey 2002; Martinón‐Torres et al. 2012; Martínez de Pinillos et al. 2014; Harvati 2015; Scott et al. 2018). Although the presence of this trait is not exclusive to Neanderthals (Martinón‐Torres et al. 2008; Scott et al. 2018; Demeter et al. 2022; Martínez de Pinillos et al. 2017), Neanderthal groups characteristically exhibit very high frequencies of MdTC. A study by Martínez de Pinillos et al. (2014) found that, at the OES, a continuous MdTC is present in 93% of early Neanderthals (Sima de los Huesos), 97% of late Neanderthals, and 39.2% of modern humans (based on total molar samples). At the EDJ, the early and late Neanderthal lower molar samples exhibit slightly reduced frequencies (72% and 76.4%, respectively) since a portion of cases observed as MdTC at OES were reclassified as MeTC (Martínez de Pinillos et al. 2014). Based on the presence of a continuous MdTC in the two M_3_ specimens of *Australopithecus africanus*, Bailey et al. (2011) suggested that the presence of this feature at the EDJ in Neanderthals may represent a retained primitive condition, while its lack could be a derived condition in the genus *Homo*. Whatever the phylogenetic polarity of the trait, the presence of a continuous MdTC in BH‐15 can be understood as a Neanderthal‐like morphological signal. With the combination of a continuous MdTC and a discontinuous DTC—with its lingual origin set within the distal cusp segment—and the pronounced distal ridge present on the buccal side, the trigonid crest pattern observed on the dentine surface of BH‐15 aligns with type 10 of the 14 different trigonid crest configurations at the EDJ defined by Martínez de Pinillos et al. (2014). Within the four‐grade system proposed by Bailey et al. (2011: 509), a Grade 2 trigonid crest is defined as the “presence of a crest whose height dips and/or is much reduced at the sagittal sulcus but remains continuous”—which aptly characterizes the expression of MdTC in the EDJ of the BH‐15 specimen. Given the geographical provenance and chronology, along with the contextual association to previously published dental remains from Velika Balanica identified as early Neanderthal, the presence of a continuous MdTC in BH‐15 aligns well with a Neanderthal taxonomic interpretation.

Molar root morphology is often overlooked in paleoanthropology, and the morphology of the root of BH‐15 warrants some discussion. Taurodontism, as defined by Keith (1913), describes multi‐rooted teeth (i.e., molars and premolars) in which the furcation is displaced apically, resulting in an elongated root body and internal pulp chamber and concomitant foreshortening of the individual root branches. The stout root body of BH‐15 would be considered *hypertaurodont* under the three‐category classification scheme of Shaw (1928), which has been adopted by more recent methods (e.g., Shifman and Chanannel 1978), despite the general understanding that the ratio of root body/pulp height to total root length varies metrically within populations (Blumberg et al. 1971). Taurodontism has been associated with Neanderthals since the term was introduced by Keith (1913), and more recent studies have confirmed that Neanderthal molars are characterized by high “bifurcation indices” (Kupczik and Hublin 2010). However, because modern humans can also express high degrees of taurodontism (Jafarzadeh et al. 2008), its utility in taxonomic assessment has been limited. Taurodontism is also known to increase distally along the molar row, such that first molars typically express relatively short root bodies, while the root body length tends to be greatest in third molars—thus, the interpretation of BH‐15 as a third molar is consistent with its extreme expression of taurodontism. Beyond the furcation, the root branches of BH‐15 are also characterized by *root fusion* or, more correctly, the developmental contiguity of adjacent root branches. The roots of BH‐15 are contiguous across their buccal side, a morphology which has been described as “C‐shaped” due to its appearance in cross‐section (Cooke and Cox 1979). Fused molar roots are also known to be common in Neanderthals (Kupczik and Hublin 2010). Yet, due to their variable expression in recent human populations (Jafarzadeh and Wu 2007), their utility in taxonomic assessment has been likewise limited. BH‐15's C‐shaped roots actually represent an unusual morphology, in which, while the buccal branch of the mesial root remains contiguous with the distal root in a “C” shaped cross‐section, the mesiolingual root branch tapers to a free apex; this morphology seems to be somewhat unique and is not accounted for in typical discussions of lower molar fusion in the literature.

The severe antemortem fracture (Grade 3 *sensu* Belcastro et al. 2018) observed in the BH‐15 crown was likely caused by mechanical trauma during masticatory or non‐masticatory activities—chewing on hard food items (some nuts and seeds), biting onto a foreign object present in food (e.g., sand/grit), food processing (e.g., bone splinters accidentally generated while attempting to separate meat from the bone or access the marrow), or gripping non‐dietary items with the teeth while producing or modifying tools (Bonfiglioli et al. 2004; Lucas et al. 2008; Scott and Winn 2011; Belcastro et al. 2018). Among extant non‐human primates, species considered habitual hard‐object feeders—such as mandrills (*Mandrillus* sp.), sakis (*Pithecia* sp.), sooty mangabeys ( *Cercocebus atys* ), and Raffles' banded langurs ( *Presbytis femoralis* )—exhibit higher rates of tooth chipping (Towle and Loch 2021). Antemortem dental crown chipping is well‐documented in both the archaeological (e.g., Belcastro et al. 2007; Scott and Winn 2011; Rubio Salvador et al. 2025) and paleoanthropological records (e.g., Towle et al. 2017; Constantino and Konow 2021; Belcastro et al. 2018). While agriculturalist groups mostly exhibit higher prevalences of chipping in incisors, hunter‐gatherers show a molar‐dominant chipping pattern (Scott and Winn 2011; Rubio Salvador et al. 2025 and references therein). Among ancient hominin species, *Homo naledi* exhibits very high rates of dental chipping (44.4% of permanent teeth affected), particularly on the posterior teeth and interproximal areas (Towle et al. 2017). Regarding Neanderthals, Belcastro et al. (2018) found high frequencies of tooth fractures in both adults (47%) and subadults (26.3%) within the extensive Krapina dental sample. Moreover, chipping at the interproximal position—also displayed in the BH‐15 specimen—was found in 33.3% of posterior mandibular teeth (Belcastro et al. 2018: Table 2).

BH‐15 is also notable for its expression of pulp stones. These calcified bodies within the dental pulp are known to occur in contemporary human populations (e.g., Goga et al. 2008; da Silva et al. 2017; Ivanauskaitė et al. 2021) and archaeological modern human dental samples (e.g., Tomczyk et al. 2014, 2017, 2020; Nicklisch et al. 2021 and references therein) but have also been reported in Neanderthals (e.g., Vandermeersch et al. 1994; Garralda and Vandermeersch 2000; Garralda et al. 2020, 2022). Various causative factors have been discussed in the literature—aging (Goga et al. 2008; Udoye and Sede 2011), irritants such as trauma, periodontal disease, or caries (Braut et al. 2003; Udoye and Sede 2011; da Silva et al. 2017; Tomczyk et al. 2020), genetic predisposition and disease (VanDenBerghe et al. 1999; Kantaputra et al. 2002), cardiovascular disease (Edds et al. 2005), calcifying nanoparticles (Zeng et al. 2011), dietary habits (Bahetwar et al. 2012; Tomczyk et al. 2014)—but the etiology of pulp stones remains unknown to date (Goga et al. 2008). Reported prevalence rates vary considerably across populations, ranging from as low as 4% to as high as 100% (Goga et al. 2008 and references therein; Nicklisch et al. 2021 and references therein). Although they can be found in individuals of all age groups, the prevalence of pulp stones appears to increase with age (Goga et al. 2008; Gulsahi et al. 2009; Al‐Nazhan and Al‐Shamrani 2011; Udoye and Sede 2011). Compared to premolars and incisors, molars (both upper and lower) exhibit a significantly higher prevalence of pulp stones (Gulsahi et al. 2009; da Silva et al. 2017). Yang et al. (2015) found that pulp stones were more extensive in the molars of *WNT10a* mutant mice, which are also characterized by taurodontism, suggesting a possible genetic link between pulp stones and root morphology. To our knowledge, the BH‐15 molar represents the first record of pulp stones in Balkan non‐modern fossil hominins. Notably, a severe crown fracture on BH‐15 could be linked to the traumatic origin of the pulp stone formation in this specimen.

The new material described in this paper aligns with previous findings (Roksandic et al. 2022), further strengthening the evidence for an early Neanderthal presence at Velika Balanica around 300 ka. While it remains possible that Neanderthals were present in the region even earlier, this material represents the earliest securely dated evidence of their dispersal into the Balkans—and arguably the broader Eastern Mediterranean region (Roksandic et al. 2018). The Middle Pleistocene hominin record in the Balkans is notably complex, reflecting the presence of multiple lineages (Roksandic et al. 2023). With a minimum age between 397 and 525 ka (Rink et al. 2013), the BH‐1 partial mandible from Mala Balanica represents the earliest known hominin fossil from the Balkans and exhibits primitive, non‐Neanderthal morphology (Roksandic et al. 2011; Skinner et al. 2016) at a time when early representatives of the Neanderthal lineage were already present in Western Europe (Arsuaga et al. 2014; Zanolli et al. 2018). In addition to the dental material from Velika Balanica, Middle Pleistocene Neanderthal remains are known from the ~130 ka deposits at Krapina, Croatia (Rink et al. 1995), and from around 170 ka at Apidima, Greece (Harvati et al. 2019). Moreover, Apidima may also evidence the earliest Out‐of‐Africa presence of anatomically modern *H. sapiens* at more than 210 ka (Harvati et al. 2019). One of the best‐known Middle Pleistocene hominin fossils from Greece, the Petralona cranium—possibly dating to around 250 ka, though its age remains uncertain—exhibits some Neanderthal‐like facial traits alongside the overall similarities to African Middle Pleistocene specimens such as Kabwe 1 (Freidline et al. 2012; Harvati 2016), leaving its taxonomic status unresolved (Roksandic et al. 2023). Outside of the Balkans and Europe, Neanderthals are documented at Karain, Türkiye, dated between 250 and 200 ka (Otte et al. 1998). Neanderthal‐like features have been observed in dental remains from Qesem Cave, Israel, recovered from the stratigraphic sequence spanning 400–200 ka—though this material cannot be conclusively attributed to Neanderthals (Hershkovitz et al. 2011, 2016; Weber et al. 2016). Overall, many Middle Pleistocene hominin fossils from the region exhibit mosaic morphologies, complicating their attribution to a single lineage (Roksandic et al. 2018; Hershkovitz et al. 2021). Furthermore, dental remains from Velika Balanica are found in the context of a Yabrudian‐like lithic assemblage (Mihailović, Kuhn, et al. 2022), indicating population interactions across the Eastern Mediterranean at 300 ka. This further reinforces the notion of the Balkans as an important corridor of movement and the Eastern Mediterranean as an area of interaction between different hominin lineages. Any new find from the area is therefore relevant and demands careful consideration.

## 5 Conclusion

The morphological and metric analyses of the hominin dental material described in this paper (BH‐7, BH‐8, BH‐15, and BH‐16), together with the previously published dentition (BH‐2, BH‐3, BH‐4, and BH‐5), indicate the presence of an early Neanderthal population at the Velika Balanica cave (Roksandic et al. 2022). This is the second Neanderthal fossil‐bearing site in Serbia. Layer 4b of the cave site of Pešturina, dated to 111 ± 11 ka (Mihailović, Milošević, et al. 2022), yielded a Neanderthal molar, Pes‐3 (Radović et al. 2019), which preserved the oldest known hominin oral microbiome in its dental calculus (Fellows Yates et al. 2021). While the crown dimensions of molars from Velika Balanica align with the early Neanderthal sample from Sima de los Huesos, some measurements of the incisors (labiolingual crown diameter in BH‐8) are more similar to late Neanderthals. This observation, while interesting, is based on very few measurements and should be taken with reserve. Overall, these four newly described teeth contribute to the growing fossil evidence for Neanderthal presence in the Central Balkans during the Middle Pleistocene, approximately 300,000 years ago.

## Author Contributions

**Predrag Radović:** conceptualization, data curation (equal), formal analysis, investigation (lead), project administration (lead), methodology (equal), validation, visualization (lead), writing – original draft (lead), writing – review and editing (lead), resources (equal). **Joshua Lindal:** data curation (equal), investigation (supporting), methodology (equal), visualization (supporting), writing – original draft (supporting), writing – review and editing (supporting). **Petar Milovanović:** data curation (equal), resources (equal). **Dušan Mihailović:** funding acquisition (equal), investigation (supporting), writing – original draft (supporting), writing – review and editing (supporting). **Mirjana Roksandic:** funding acquisition (equal), investigation (supporting), project administration (supporting), supervision, writing – original draft (supporting), writing – review and editing (supporting), resources (equal).

## Conflicts of Interest

The authors declare no conflicts of interest.

## Supporting information


**Table S1:** Mesiodistal (MD) and labiolingual (LaL) crown diameters (in mm) of the BH‐8 upper central incisor from Velika Balanica (in bold) and comparative Neanderthal and modern human specimens. When both antimeres were preserved, preference was given to the right one. Abbreviations: ENEA = early Neanderthals; L = left; LNEA = late Neanderthals; MPHS = Middle Paleolithic *Homo sapiens* ; R = right; UPHS = Upper Paleolithic *H. sapiens* .
**Table S2:** Labiolingual (LaL) crown diameters (in mm) of the BH‐7 lower lateral incisor from Velika Balanica (in bold) and comparative Neanderthal and modern human specimens. When both antimeres were preserved, preference was given to the right one. Abbreviations: ENEA = early Neanderthals; L = left; LNEA = late Neanderthals; MPHS = Middle Paleolithic *Homo sapiens* ; R = right; UPHS = Upper Paleolithic *H. sapiens* .
**Table S3:** Buccolingual (BL) crown diameters (in mm) of the BH‐15 lower third molar from Velika Balanica (in bold) and comparative Neanderthal and modern human specimens. When both antimeres were preserved, preference was given to the left one. Abbreviations: ENEA = early Neanderthals; L = left; LNEA = late Neanderthals; MPHS = Middle Paleolithic *Homo sapiens* ; R = right; UPHS = Upper Paleolithic *H. sapiens* .
**Table S4:** Crown and root diameters (in mm) of BH‐5, BH‐8, BH‐7, and BH‐15 compared with Neanderthal and modern human samples. Adjusted Z‐score values above +1 or below −1, representing those outside the 95% confidence interval of the comparative group's variability, are highlighted in bold. For crown diameters, the comparative data are provided in Tables S1–S3. For root lengths, the data are sourced from Le Cabec et al. (2013, Table 4b) (original group names: “Neanderthals,” “Early modern humans,” “Upper Paleolithic and Epipaleolithic humans,” and “Recent modern humans”), except for early Neanderthal (i.e., Sima de los Huesos) I_2_s, which come from Lockey et al. (2023, Table 4). Abbreviations: BL = buccolingual crown diameter; LaL = labiolingual crown diameter; MD = mesiodistal crown diameter; *n* = number of specimens per sample; RL = root length; SD = standard deviation.

## Data Availability

The fossil specimens described in this study are currently stored at the Center for Paleolithic and Mesolithic Research, Faculty of Philosophy, University of Belgrade. The μCT scans of the fossils are stored on a server at the University of Belgrade and are available upon request from M. Roksandic and D. Mihailović.
