# Supplementary material for: New Middle Pleistocene Hominin Dental Remains From Velika Balanica, Serbia
Source: Am J Biol Anthropol. 2025 Oct 6;188(2):e70133. doi: 10.1002/ajpa.70133 (PMC12498494; doi:10.1002/ajpa.70133)
Supplement: Supplementary file 1 — Table S1: Mesiodistal (MD) and labiolingual (LaL) crown diameters (in mm) of the BH‐8 upper central incisor from Velika Balanica (in bold) and comparative Neanderthal and modern human specimens. When both antimeres were preserved, preference was given to the right one. Abbreviations: ENEA = early Neanderthals; L = left; LNEA = late Neanderthals; MPHS = Middle Paleolithic Homo sapiens ; R = right; UPHS = Upper Paleolithic H. sapiens . Table S2: Labiolingual (LaL) crown diameters (in mm) of the BH‐7 lower lateral incisor from Velika Balanica (in bold) and comparative Neanderthal and modern human specimens. When both antimeres were preserved, preference was given to the right one. Abbreviations: ENEA = early Neanderthals; L = left; LNEA = late Neanderthals; MPHS = Middle Paleolithic Homo sapiens ; R = right; UPHS = Upper Paleolithic H. sapiens . Table S3: Buccolingual (BL) crown diameters (in mm) of the BH‐15 lower third molar from Velika Balanica (in bold) and comparative Neanderthal and modern human specimens. When both antimeres were preserved, preference was given to the left one. Abbreviations: ENEA = early Neanderthals; L = left; LNEA = late Neanderthals; MPHS = Middle Paleolithic Homo sapiens ; R = right; UPHS = Upper Paleolithic H. sapiens . Table S4: Crown and root diameters (in mm) of BH‐5, BH‐8, BH‐7, and BH‐15 compared with Neanderthal and modern human samples. Adjusted Z‐score values above +1 or below −1, representing those outside the 95% confidence interval of the comparative group's variability, are highlighted in bold. For crown diameters, the comparative data are provided in Tables S1–S3. For root lengths, the data are sourced from Le Cabec et al. (2013, Table 4b) (original group names: “Neanderthals,” “Early modern humans,” “Upper Paleolithic and Epipaleolithic humans,” and “Recent modern humans”), except for early Neanderthal (i.e., Sima de los Huesos) I2s, which come from Lockey et al. (2023, Table 4). Abbreviations: BL = buccolingual crown diamete [file AJPA-188-e70133-s001.docx]

Supplementary Material for

**New Middle Pleistocene hominin dental remains from Velika Balanica, Serbia**

Predrag Radović, Joshua Lindal, Petar Milovanović, Dušan Mihailović, Mirjana Roksandic

Correspondence: Predrag Radović (predrag.radovic@f.bg.ac.rs), Mirjana Roksandic (m.roksandic@uwinnipeg.ca)

**TABLE S1.** Mesiodistal (MD) and labiolingual (LaL) crown diameters (in mm) of the BH-8 upper central incisor from Velika Balanica (in bold) and comparative Neanderthal and modern human specimens. When both antimeres were preserved, preference was given to the right one. Abbreviations: ENEA = early Neanderthals; L = left; LNEA = late Neanderthals; MPHS = Middle Paleolithic *Homo sapiens*; R = right; UPHS = Upper Paleolithic *H. sapiens*.

| Country | Site | Specimen | Group | Tooth | Side | MD | LaL | Source |
| --- | --- | --- | --- | --- | --- | --- | --- | --- |
| **Serbia** | **Velika Balanica** | **BH-8** | – | **I^1^** | **R** | **10.2** | **8.8** | **This study** |
| Spain | Sima de los Huesos | AT-42 | ENEA | I^1^ | R | 9.6 | 7.7 | Martinón-Torres et al. (2012) |
| Spain | Sima de los Huesos | AT-553 | ENEA | I^1^ | R | 8.1 | 8.1 | Martinón-Torres et al. (2012) |
| Spain | Sima de los Huesos | AT-560 | ENEA | I^1^ | L | 9.6 | 7.8 | Martinón-Torres et al. (2012) |
| Spain | Sima de los Huesos | AT-2786 | ENEA | I^1^ | R | 9.0 | 7.4 | Martinón-Torres et al. (2012) |
| Spain | Sima de los Huesos | AT-2395 | ENEA | I^1^ | R | 10.2 | 7.6 | Martinón-Torres et al. (2012) |
| Spain | Sima de los Huesos | AT-954 | ENEA | I^1^ | R | 10.6 | 8.0 | Martinón-Torres et al. (2012) |
| Spain | Sima de los Huesos | AT-814 | ENEA | I^1^ | R | 9.1 | 7.6 | Martinón-Torres et al. (2012) |
| Spain | Sima de los Huesos | AT-4320 | ENEA | I^1^ | L | 9.3 | 7.1 | Martinón-Torres et al. (2012) |
| Spain | Sima de los Huesos | AT-198 | ENEA | I^1^ | R | 9.7 | 8.0 | Martinón-Torres et al. (2012) |
| Spain | Sima de los Huesos | AT-8 | ENEA | I^1^ | L | 10.0 | 8.0 | Martinón-Torres et al. (2012) |
| Spain | Sima de los Huesos | AT-54 | ENEA | I^1^ | R | 9.9 | 7.5 | Martinón-Torres et al. (2012) |
| Spain | Sima de los Huesos | AT-146 | ENEA | I^1^ | R | 9.5 | 7.7 | Martinón-Torres et al. (2012) |
| Spain | Sima de los Huesos | AT-197 | ENEA | I^1^ | R | 9.2 | 7.5 | Martinón-Torres et al. (2012) |
| Spain | Sima de los Huesos | AT-278 | ENEA | I^1^ | R | 9.4 | 7.7 | Martinón-Torres et al. (2012) |
| Spain | Sima de los Huesos | AT-280 | ENEA | I^1^ | L | 9.4 | 7.9 | Martinón-Torres et al. (2012) |
| Spain | Sima de los Huesos | AT-1943 | ENEA | I^1^ | R | 9.5 | 7.9 | Martinón-Torres et al. (2012) |
| Spain | Sima de los Huesos | AT-1958 | ENEA | I^1^ | L | 9.2 | 7.6 | Martinón-Torres et al. (2012) |
| Spain | Sima de los Huesos | AT-3194 | ENEA | I^1^ | L | 9.6 | 7.8 | Martinón-Torres et al. (2012) |
| Spain | Sima de los Huesos | AT-5615 | ENEA | I^1^ | R | 8.7 | 7.3 | Martinón-Torres et al. (2012) |
| Spain | Sima de los Huesos | AT-3885 | ENEA | I^1^ | L | 9.0 | 8.8 | Martinón-Torres et al. (2012) |
| Belgium | Scladina | Scla 4A-11 | LNEA | I^1^ | R | 9.9 | 7.98 | Voisin et al. (2012) |
| Croatia | Krapina | 92 | LNEA | I^1^ | R | 10.9 | 9.4 | Voisin et al. (2012) |
| Croatia | Krapina | D/D | LNEA | I^1^ | R | 10.0 | 8.8 | Voisin et al. (2012) |
| Croatia | Krapina | F/H | LNEA | I^1^ | R | 9.4 | 8.9 | Voisin et al. (2012) |
| Croatia | Krapina | MX E | LNEA | I^1^ | R | 10.0 | 8.3 | Voisin et al. (2012) |
| Croatia | Krapina | MX H | LNEA | I^1^ | L | 10.7 | 9.5 | Voisin et al. (2012) |
| Croatia | Krapina | MX K | LNEA | I^1^ | R | 10.5 | 9.3 | Voisin et al. (2012) |
| Croatia | Krapina | MX Q | LNEA | I^1^ | R | 10.0 | 8.8 | Voisin et al. (2012) |
| Croatia | Krapina | N/N | LNEA | I^1^ | R | 10.9 | 9.7 | Voisin et al. (2012) |
| France | La Chaise-de-Vouthon | BD12 | LNEA | I^1^ | – | 8.2 | 8.4 | Voisin et al. (2012) |
| France | Marillac | M13A | LNEA | I^1^ | R | 9.9 | 7.92 | Voisin et al. (2012) |
| France | Monsempron | Pièce c | LNEA | I^1^ | – | 9 | 8.5 | Voisin et al. (2012) |
| France | Vergisson | Vergisson 4-83 | LNEA | I^1^ | L | 12.3 | 9.6 | Voisin et al. (2012) |
| Germany | Neanderthal | NN66 | LNEA | I^1^ | R | 7.8 | 8.6 | Voisin et al. (2012) |
| Iraq | Shanidar | Shanidar 2 | LNEA | I^1^ | L | 8.2 | 8.2 | Voisin et al. (2012) |
| Israel | Amud | Amud 1 | LNEA | I^1^ | R | 9.2 | 8.2 | Voisin et al. (2012) |
| Spain | Carihuela Cave | Pinar 7 | LNEA | I^1^ | L | 10.6 | 9.0 | Voisin et al. (2012) |
| Spain | Cova Negra | C.N. 7856 | LNEA | I^1^ | R | 8.5 | 7.8 | Voisin et al. (2012) |
| Spain | Palomas | Palomas 24 | LNEA | I^1^ | L | 9.1 | 9.3 | Voisin et al. (2012) |
| Spain | Palomas | Palomas 34 | LNEA | I^1^ | L | 9.2 | 8.0 | Voisin et al. (2012) |
| Israel | Qafzeh | Qafzeh 5 | MPHS | I^1^ | R | 9.0 | 7.3 | Voisin et al. (2012) |
| Israel | Qafzeh | Qafzeh 7 | MPHS | I^1^ | R | 9.2 | 8.7 | Voisin et al. (2012) |
| Israel | Qafzeh | Qafzeh 9 | MPHS | I^1^ | L | 11.1 | 8.2 | Voisin et al. (2012) |
| Czechia | Dolní Vestonice | DV13 | UPHS | I^1^ | L | 9.3 | 8.2 | Voisin et al. (2012) |
| Czechia | Dolní Vestonice | DV14 | UPHS | I^1^ | R | 9.8 | 8.0 | Voisin et al. (2012) |
| Czechia | Dolní Vestonice | DV15 | UPHS | I^1^ | R | 8.7 | 7.6 | Voisin et al. (2012) |
| Czechia | Předmostí | 1 | UPHS | I^1^ | L | 9.0 | 7.8 | Voisin et al. (2012) |
| Czechia | Předmostí | 3 | UPHS | I^1^ | R | 8.0 | 8.0 | Voisin et al. (2012) |
| Czechia | Předmostí | 4 | UPHS | I^1^ | L | 8.8 | 7.3 | Voisin et al. (2012) |
| Czechia | Předmostí | 5 | UPHS | I^1^ | L | 9.0 | 7.4 | Voisin et al. (2012) |
| Czechia | Předmostí | 9 | UPHS | I^1^ | R | 9.1 | 7.4 | Voisin et al. (2012) |
| Czechia | Předmostí | 10 | UPHS | I^1^ | R | 9.0 | 7.3 | Voisin et al. (2012) |
| Czechia | Předmostí | 14 | UPHS | I^1^ | R | 9.0 | 7.5 | Voisin et al. (2012) |
| France | Abri Pataud | P1 | UPHS | I^1^ | R | 8.6 | 7.8 | Voisin et al. (2012) |
| France | Abri Pataud | 2.2088 | UPHS | I^1^ | L | 8.2 | 6.9 | Voisin et al. (2012) |
| France | Cap Balanc | 1 | UPHS | I^1^ | R | 7.2 | 7.0 | Voisin et al. (2012) |
| France | Le Peyrat | Le Peyrat 5 | UPHS | I^1^ | R | 5.7 | 6.6 | Voisin et al. (2012) |
| France | Les Rois | R50.21 | UPHS | I^1^ | L | 9.6 | 7.7 | Voisin et al. (2012) |
| France | Les Rois | R51.45 | UPHS | I^1^ | L | 10.2 | 7.3 | Voisin et al. (2012) |
| France | Les Rois | 55.148f | UPHS | I^1^ | R | 10.7 | 7.8 | Voisin et al. (2012) |
| France | Les Rois | 55.148d | UPHS | I^1^ | R | 10.8 | 7.8 | Voisin et al. (2012) |
| France | St. Germain La Rivière | 12 | UPHS | I^1^ | L | 9.0 | 7.7 | Voisin et al. (2012) |
| France | St. Germain La Rivière | 15 | UPHS | I^1^ | L | 9.0 | 7.6 | Voisin et al. (2012) |
| France | St. Germain La Rivière | 16 | UPHS | I^1^ | R | 9.5 | 7.4 | Voisin et al. (2012) |
| Italy | Arene Candide | 1 | UPHS | I^1^ | R | 9.5 | 8.3 | Voisin et al. (2012) |
| Italy | Barma Grande | 3 | UPHS | I^1^ | L | 9.3 | 7.6 | Voisin et al. (2012) |
| Italy | Grotte des Enfants | 6 | UPHS | I^1^ | R | 10.1 | 8.1 | Voisin et al. (2012) |
| Italy | Paglicci | 12 | UPHS | I^1^ | R | 9.2 | 7.8 | Voisin et al. (2012) |

**TABLE S2.** Labiolingual (LaL) crown diameters (in mm) of the BH-7 lower lateral incisor from Velika Balanica (in bold) and comparative Neanderthal and modern human specimens. When both antimeres were preserved, preference was given to the right one. Abbreviations: ENEA = early Neanderthals; L = left; LNEA = late Neanderthals; MPHS = Middle Paleolithic *Homo sapiens*; R = right; UPHS = Upper Paleolithic *H. sapiens*.

| Country | Site | Specimen | Group | Tooth | Side | LaL | Source |
| --- | --- | --- | --- | --- | --- | --- | --- |
| **Serbia** | **Velika Balanica** | **BH-7** | **–** | **I_2_** | **R** | **8.1** | **This study** |
| Italy | Fontana Ranuccio | FR2 | ENEA | I_2_ | L | 7.7 | Voisin et al. (2012) |
| UK | Pontnewydd | PN10 | ENEA | I_2_ | R | 6.8 | Voisin et al. (2012) |
| Spain | Sima de los Huesos | AT-275 | ENEA | I_2_ | R | 7.4 | Martinón-Torres et al. (2012) |
| Spain | Sima de los Huesos | AT-55 | ENEA | I_2_ | R | 7.3 | Martinón-Torres et al. (2012) |
| Spain | Sima de los Huesos | AT-1726 | ENEA | I_2_ | R | 7.1 | Martinón-Torres et al. (2012) |
| Spain | Sima de los Huesos | AT-5 | ENEA | I_2_ | R | 8.1 | Martinón-Torres et al. (2012) |
| Spain | Sima de los Huesos | AT-723 | ENEA | I_2_ | R | 6.7 | Martinón-Torres et al. (2012) |
| Spain | Sima de los Huesos | AT-300 | ENEA | I_2_ | R | 7.6 | Martinón-Torres et al. (2012) |
| Spain | Sima de los Huesos | AT-1461 | ENEA | I_2_ | R | 7.2 | Martinón-Torres et al. (2012) |
| Spain | Sima de los Huesos | AT-3256 | ENEA | I_2_ | R | 7.2 | Martinón-Torres et al. (2012) |
| Spain | Sima de los Huesos | AT-957 | ENEA | I_2_ | R | 6.8 | Martinón-Torres et al. (2012) |
| Spain | Sima de los Huesos | AT-1123 | ENEA | I_2_ | R | 8.0 | Martinón-Torres et al. (2012) |
| Spain | Sima de los Huesos | AT-2753 | ENEA | I_2_ | R | 7.9 | Martinón-Torres et al. (2012) |
| Spain | Sima de los Huesos | AT-594 | ENEA | I_2_ | R | 7.3 | Martinón-Torres et al. (2012) |
| Spain | Sima de los Huesos | AT-281 | ENEA | I_2_ | R | 7.1 | Martinón-Torres et al. (2012) |
| Spain | Sima de los Huesos | AT-3827 | ENEA | I_2_ | R | 7.1 | Martinón-Torres et al. (2012) |
| Spain | Sima de los Huesos | AT-2776 | ENEA | I_2_ | L | 6.6 | Martinón-Torres et al. (2012) |
| Spain | Sima de los Huesos | AT-282 | ENEA | I_2_ | R | 7.4 | Martinón-Torres et al. (2012) |
| Spain | Sima de los Huesos | AT-2278 | ENEA | I_2_ | L | 7.4 | Martinón-Torres et al. (2012) |
| Spain | Sima de los Huesos | AT-592 | ENEA | I_2_ | L | 7.1 | Martinón-Torres et al. (2012) |
| Belgium | Scladina | Scla 4A-20 | LNEA | I_2_ | R | 7.28 | Voisin et al. (2012) |
| Croatia | Krapina | D/D | LNEA | I_2_ | L | 7.3 | Voisin et al. (2012) |
| Croatia | Krapina | F/H | LNEA | I_2_ | R | 7.8 | Voisin et al. (2012) |
| Croatia | Krapina | MND C | LNEA | I_2_ | L | 8.8 | Voisin et al. (2012) |
| Croatia | Krapina | MND E | LNEA | I_2_ | L | 8.3 | Voisin et al. (2012) |
| Croatia | Krapina | MND J | LNEA | I_2_ | R | 8.0 | Voisin et al. (2012) |
| Croatia | Krapina | MND O | LNEA | I_2_ | R | 8.8 | Voisin et al. (2012) |
| Croatia | Krapina | MND L | LNEA | I_2_ | R | 8.1 | Voisin et al. (2012) |
| France | La Chaise-de-Vouthon | BD1 | LNEA | I_2_ | R | 7.6 | Voisin et al. (2012) |
| France | La Chaise-de-Vouthon | BD21 | LNEA | I_2_ | L | 6.8 | Voisin et al. (2012) |
| France | Montgaudier | Montgaudier | LNEA | I_2_ | L | 6.9 | Voisin et al. (2012) |
| France | Regourdou | Regourdou 1 | LNEA | I_2_ | R | 7.56 | Voisin et al. (2012) |
| France | Grotte du Renne | 11 | LNEA | I_2_ | L | 7.8 | Voisin et al. (2012) |
| Hungary | Subalyuk | Subalyuk 1 | LNEA | I_2_ | R | 8.1 | Voisin et al. (2012) |
| Iraq | Shanidar | Shanidar 1 | LNEA | I_2_ | R | 8.5 | Voisin et al. (2012) |
| Iraq | Shanidar | Shanidar 2 | LNEA | I_2_ | R | 8.6 | Voisin et al. (2012) |
| Iraq | Shanidar | Shanidar 4 | LNEA | I_2_ | R | 8.4 | Voisin et al. (2012) |
| Israel | Kebara | Kebara 2 | LNEA | I_2_ | R | 8.1 | Voisin et al. (2012) |
| Spain | Palomas | Palomas 1 | LNEA | I_2_ | L | 6.8 | Voisin et al. (2012) |
| Spain | Palomas | Palomas 89 | LNEA | I_2_ | R | 7.2 | Voisin et al. (2012) |
| Spain | Palomas | Palomas 91 | LNEA | I_2_ | L | 7.8 | Voisin et al. (2012) |
| Spain | Valdegoba | Valdegoba 1 | LNEA | I_2_ | R | 7.8 | Voisin et al. (2012) |
| Italy | Arma delle Mànie | Le Mànie 1 | LNEA | I_2_ | – | 8.6 | Voisin et al. (2012) |
| Israel | Qafzeh | Qafzeh 7 | MPHS | I_2_ | L | 7.1 | Voisin et al. (2012) |
| Israel | Qafzeh | Qafzeh 8 | MPHS | I_2_ | R | 6.7 | Voisin et al. (2012) |
| Israel | Qafzeh | Qafzeh 9 | MPHS | I_2_ | L | 7.8 | Voisin et al. (2012) |
| Morocco | Jebel Irhoud | Irhoud 11 | MPHS | I_2_ | R | 7.4 | Voisin et al. (2012) |
| Bulgaria | Bacho Kiro | 2641 | UPHS | I_2_ | R | 7.3 | Voisin et al. (2012) |
| Czechia | Dolní Vestonice | DV3 | UPHS | I_2_ | R | 6.62 | Voisin et al. (2012) |
| Czechia | Dolní Vestonice | DV13 | UPHS | I_2_ | R | 8.1 | Voisin et al. (2012) |
| Czechia | Dolní Vestonice | DV14 | UPHS | I_2_ | R | 7.0 | Voisin et al. (2012) |
| Czechia | Dolní Vestonice | DV15 | UPHS | I_2_ | R | 6.9 | Voisin et al. (2012) |
| Czechia | Mladeč | 54 | UPHS | I_2_ | R | 7.5 | Voisin et al. (2012) |
| Czechia | Pavlov | 1 | UPHS | I_2_ | R | 6.6 | Voisin et al. (2012) |
| Czechia | Pavlov | 592256.84 | UPHS | I_2_ | L | 5.9 | Voisin et al. (2012) |
| Czechia | Pavlov | 519156 | UPHS | I_2_ | R | 6.1 | Voisin et al. (2012) |
| Czechia | Pavlov | 641436 | UPHS | I_2_ | L | 5.7 | Voisin et al. (2012) |
| Czechia | Předmostí | 3 | UPHS | I_2_ | L | 7.0 | Voisin et al. (2012) |
| Czechia | Předmostí | 4 | UPHS | I_2_ | R | 6.0 | Voisin et al. (2012) |
| Czechia | Předmostí | 5 | UPHS | I_2_ | R | 7.0 | Voisin et al. (2012) |
| Czechia | Předmostí | 7 | UPHS | I_2_ | R | 6.7 | Voisin et al. (2012) |
| Czechia | Předmostí | 9 | UPHS | I_2_ | R | 7.0 | Voisin et al. (2012) |
| Czechia | Předmostí | 10 | UPHS | I_2_ | R | 6.0 | Voisin et al. (2012) |
| Czechia | Předmostí | 14 | UPHS | I_2_ | L | 7.0 | Voisin et al. (2012) |
| Czechia | Předmostí | 18 | UPHS | I_2_ | L | 7.0 | Voisin et al. (2012) |
| Czechia | Předmostí | 20 | UPHS | I_2_ | L | 7.0 | Voisin et al. (2012) |
| France | Cap Blanc | 1 | UPHS | I_2_ | R | 6.1 | Voisin et al. (2012) |
| France | Lachaud | – | UPHS | I_2_ | R | 6.7 | Voisin et al. (2012) |
| France | Le Peyrat | Le Peyrat 5 | UPHS | I_2_ | R | 6.6 | Voisin et al. (2012) |
| France | Les Rois | R51.17 | UPHS | I_2_ | L | 6.9 | Voisin et al. (2012) |
| France | Les Rois | R50.24 | UPHS | I_2_ | L | 7.2 | Voisin et al. (2012) |
| France | Les Rois | 55.148g | UPHS | I_2_ | R | 7.5 | Voisin et al. (2012) |
| France | Grotte de la Balauzière | L.B. II | UPHS | I_2_ | L | 5.8 | Voisin et al. (2012) |
| France | St. Germain La Rivière | B31970.8 | UPHS | I_2_ | R | 6.6 | Voisin et al. (2012) |
| France | St. Germain La Rivière | 4 | UPHS | I_2_ | L | 7.0 | Voisin et al. (2012) |
| France | St. Germain La Rivière | B41970.8 | UPHS | I_2_ | R | 6.55 | Voisin et al. (2012) |
| Israel | Neve David | ND1 | UPHS | I_2_ | L | 5.9 | Voisin et al. (2012) |
| Spain | El Mirón | El Mirón 1 | UPHS | I_2_ | R | 6.3 | Voisin et al. (2012) |
| Portugal | Gruta do Caldeirão | Caldeirão 5 | UPHS | I_2_ | R | 6.4 | Voisin et al. (2012) |
| Portugal | Gruta do Caldeirão | Caldeirão 6 | UPHS | I_2_ | R | 6.5 | Voisin et al. (2012) |
| Italy | Arene Candide | 1 | UPHS | I_2_ | R | 7.6 | Voisin et al. (2012) |
| Italy | Arene Candide | 2 | UPHS | I_2_ | R | 6.4 | Voisin et al. (2012) |
| Italy | Arene Candide | 4 | UPHS | I_2_ | R | 6.9 | Voisin et al. (2012) |
| Italy | Arene Candide | 5 | UPHS | I_2_ | R | 7.3 | Voisin et al. (2012) |
| Italy | Arene Candide | 19.6725 | UPHS | I_2_ | R | 6.5 | Voisin et al. (2012) |
| Italy | Barma Grande | 4 | UPHS | I_2_ | R | 6.6 | Voisin et al. (2012) |
| Italy | Barma Grande | 5 | UPHS | I_2_ | L | 7.5 | Voisin et al. (2012) |
| Italy | Grotte des Enfants | 4 | UPHS | I_2_ | L | 6.4 | Voisin et al. (2012) |
| Italy | Grotte des Enfants | 6 | UPHS | I_2_ | R | 7.2 | Voisin et al. (2012) |
| Italy | Paglicci | 12 | UPHS | I_2_ | R | 7.0 | Voisin et al. (2012) |
| Italy | Romito | 1 | UPHS | I_2_ | R | 5.9 | Voisin et al. (2012) |
| Italy | Romito | 3 | UPHS | I_2_ | R | 6.9 | Voisin et al. (2012) |
| Italy | Romito | 4 | UPHS | I_2_ | R | 6.5 | Voisin et al. (2012) |
| Italy | Romito | 5 | UPHS | I_2_ | R | 6.7 | Voisin et al. (2012) |
| Italy | Romito | 6 | UPHS | I_2_ | R | 6.0 | Voisin et al. (2012) |

**TABLE S3.** Buccolingual (BL) crown diameters (in mm) of the BH-15 lower third molar from Velika Balanica (in bold) and comparative Neanderthal and modern human specimens. When both antimeres were preserved, preference was given to the left one. Abbreviations: ENEA = early Neanderthals; L = left; LNEA = late Neanderthals; MPHS = Middle Paleolithic *Homo sapiens*; R = right; UPHS = Upper Paleolithic *H. sapiens*.

| Country | Site | Specimen | Group | Tooth | Side | BL | Source |
| --- | --- | --- | --- | --- | --- | --- | --- |
| **Serbia** | **Velika Balanica** | **BH-15** | – | **M_3_** | **L** | **10.2** | **This study** |
| UK | Pontnewydd | PN16 | ENEA | M_3_ | R | 10.6 | Voisin et al. (2012) |
| UK | Pontnewydd | PN21 | ENEA | M_3_ | R | 10.9 | Voisin et al. (2012) |
| Spain | Sima de los Huesos | AT-1 | ENEA | M_3_ | L | 9.2 | Martinón-Torres et al. (2012) |
| Spain | Sima de los Huesos | AT-811 | ENEA | M_3_ | L | 9.8 | Martinón-Torres et al. (2012) |
| Spain | Sima de los Huesos | AT-75 | ENEA | M_3_ | L | 9.2 | Martinón-Torres et al. (2012) |
| Spain | Sima de los Huesos | AT-13 | ENEA | M_3_ | L | 11.3 | Martinón-Torres et al. (2012) |
| Spain | Sima de los Huesos | AT-4147 | ENEA | M_3_ | L | 10.9 | Martinón-Torres et al. (2012) |
| Spain | Sima de los Huesos | AT-2193 | ENEA | M_3_ | R | 9.3 | Martinón-Torres et al. (2012) |
| Spain | Sima de los Huesos | AT-222 | ENEA | M_3_ | R | 9.8 | Martinón-Torres et al. (2012) |
| Spain | Sima de los Huesos | AT-2271 | ENEA | M_3_ | L | 9.8 | Martinón-Torres et al. (2012) |
| Spain | Sima de los Huesos | XIX | ENEA | M_3_ | L | 8.7 | Martinón-Torres et al. (2012) |
| Spain | Sima de los Huesos | AT-605 | ENEA | M_3_ | L | 10.6 | Martinón-Torres et al. (2012) |
| Spain | Sima de los Huesos | AT-607 | ENEA | M_3_ | L | 9.9 | Martinón-Torres et al. (2012) |
| Spain | Sima de los Huesos | AT-2385 | ENEA | M_3_ | L | 8.9 | Martinón-Torres et al. (2012) |
| Spain | Sima de los Huesos | AT-3943 | ENEA | M_3_ | R | 8.8 | Martinón-Torres et al. (2012) |
| Spain | Sima de los Huesos | AT-30 | ENEA | M_3_ | R | 9.7 | Martinón-Torres et al. (2012) |
| Spain | Sima de los Huesos | AT-792 | ENEA | M_3_ | L | 10.6 | Martinón-Torres et al. (2012) |
| Spain | Sima de los Huesos | AT-950 | ENEA | M_3_ | L | 9.7 | Martinón-Torres et al. (2012) |
| Spain | Sima de los Huesos | AT-598 | ENEA | M_3_ | L | 9.8 | Martinón-Torres et al. (2012) |
| Spain | Sima de los Huesos | AT-1473 | ENEA | M_3_ | L | 9.4 | Martinón-Torres et al. (2012) |
| Spain | Sima de los Huesos | AT-143 | ENEA | M_3_ | R | 10.1 | Martinón-Torres et al. (2012) |
| Spain | Sima de los Huesos | AT-2777 | ENEA | M_3_ | R | 11.0 | Martinón-Torres et al. (2012) |
| Spain | Sima de los Huesos | AT-1945 | ENEA | M_3_ | L | 9.6 | Martinón-Torres et al. (2012) |
| Spain | Sima de los Huesos | AT-2760 | ENEA | M_3_ | L | 8.4 | Martinón-Torres et al. (2012) |
| Spain | Sima de los Huesos | AT-3182 | ENEA | M_3_ | R | 9.6 | Martinón-Torres et al. (2012) |
| Spain | Sima de los Huesos | AT-942 | ENEA | M_3_ | R | 9.5 | Martinón-Torres et al. (2012) |
| Spain | Sima de los Huesos | AT-2273 | ENEA | M_3_ | L | 9.7 | Martinón-Torres et al. (2012) |
| Croatia | Krapina | 4 | LNEA | M_3_ | L | 10.32 | Voisin et al. (2012) |
| Croatia | Krapina | 5 | LNEA | M_3_ | L | 12.01 | Voisin et al. (2012) |
| Croatia | Krapina | 8 | LNEA | M_3_ | R | 11.2 | Voisin et al. (2012) |
| Croatia | Krapina | 9 | LNEA | M_3_ | L | 10.7 | Voisin et al. (2012) |
| Croatia | Krapina | 78 | LNEA | M_3_ | L | 10.4 | Voisin et al. (2012) |
| Croatia | Krapina | F/H | LNEA | M_3_ | L | 10.7 | Voisin et al. (2012) |
| Croatia | Krapina | MND E | LNEA | M_3_ | L | 11.1 | Voisin et al. (2012) |
| Croatia | Krapina | MND G | LNEA | M_3_ | R | 11.2 | Voisin et al. (2012) |
| Croatia | Krapina | MND J | LNEA | M_3_ | R | 11.3 | Voisin et al. (2012) |
| Croatia | Krapina | MND K | LNEA | M_3_ | L | 10.6 | Voisin et al. (2012) |
| Croatia | Krapina | MND M | LNEA | M_3_ | L | 11.4 | Voisin et al. (2012) |
| Croatia | Krapina | MND L | LNEA | M_3_ | L | 9.8 | Voisin et al. (2012) |
| Croatia | Vindija | 76 231 11.45 | LNEA | M_3_ | L | 11.52 | Voisin et al. (2012) |
| Croatia | Vindija | 15 206 11.39 | LNEA | M_3_ | R | 11.67 | Voisin et al. (2012) |
| France | Combe-Grenal | XII | LNEA | M_3_ | L | 11.9 | Voisin et al. (2012) |
| France | Arcy sur Cure | P7-647-76 | LNEA | M_3_ | L | 11.6 | Voisin et al. (2012) |
| France | Bourgeois Delaunay | BD1 | LNEA | M_3_ | L | 9.6 | Voisin et al. (2012) |
| France | La Ferrassie | LF 10 | LNEA | M_3_ | R | 10.3 | Voisin et al. (2012) |
| France | La Ferrassie | LF 12 | LNEA | M_3_ | R | 8.4 | Voisin et al. (2012) |
| France | La Quina | La Quina 5 | LNEA | M_3_ | L | 11.56 | Voisin et al. (2012) |
| France | La Quina | La Quina 4b | LNEA | M_3_ | L | 12.23 | Voisin et al. (2012) |
| France | La Quina | La Quina 9 | LNEA | M_3_ | L | 12.40 | Voisin et al. (2012) |
| France | Moula-Guercy | M-G2-419 | LNEA | M_3_ | R | 10.5 | Voisin et al. (2012) |
| France | Regourdou | Regourdou 1 | LNEA | M_3_ | L | 10.87 | Voisin et al. (2012) |
| Greece | Lakonis I | LKH 1 | LNEA | M_3_ | L | 10.9 | Voisin et al. (2012) |
| Hungary | Subalyuk | Subalyuk 1 | LNEA | M_3_ | L | 11.5 | Voisin et al. (2012) |
| Iraq | Shanidar | Shanidar 1 | LNEA | M_3_ | L | 10.8 | Voisin et al. (2012) |
| Iraq | Shanidar | Shanidar 2 | LNEA | M_3_ | L | 11.2 | Voisin et al. (2012) |
| Iraq | Shanidar | Shanidar 6 | LNEA | M_3_ | R | 12.2 | Voisin et al. (2012) |
| Israel | Amud | Amud 1 | LNEA | M_3_ | L | 10.8 | Voisin et al. (2012) |
| Israel | Kebara | Kebara 2 | LNEA | M_3_ | L | 11.3 | Voisin et al. (2012) |
| Israel | Me'arat Shovakh | Shovakh 1 | LNEA | M_3_ | L | 10.9 | Voisin et al. (2012) |
| Spain | Palomas | Palomas 1 | LNEA | M_3_ | R | 10.6 | Voisin et al. (2012) |
| Spain | Palomas | Palomas 58 | LNEA | M_3_ | L | 11.0 | Voisin et al. (2012) |
| Spain | Palomas | Palomas 50 | LNEA | M_3_ | R | 10.7 | Voisin et al. (2012) |
| France | Grotte du Renne | 6 | LNEA | M_3_ | R | 10.8 | Voisin et al. (2012) |
| Spain | Valdegoba | Valdegoba 1 | LNEA | M_3_ | L | 10.3 | Voisin et al. (2012) |
| Italy | Fate | F3 | LNEA | M_3_ | R | 11.0 | Voisin et al. (2012) |
| Israel | Qafzeh | Qafzeh 7 | MPHS | M_3_ | R | 11.3 | Voisin et al. (2012) |
| Israel | Qafzeh | Qafzeh 8 | MPHS | M_3_ | R | 10.9 | Voisin et al. (2012) |
| Israel | Qafzeh | Qafzeh 9 | MPHS | M_3_ | L | 11.9 | Voisin et al. (2012) |
| Morocco | Djebel Irhoud | Irhoud 11 | MPHS | M_3_ | L | 11.0 | Voisin et al. (2012) |
| Czechia | Dolní Vestonice | DV3 | UPHS | M_3_ | R | 10.5 | Voisin et al. (2012) |
| Czechia | Dolní Vestonice | DV13 | UPHS | M_3_ | L | 10.3 | Voisin et al. (2012) |
| Czechia | Dolní Vestonice | DV15 | UPHS | M_3_ | L | 11.3 | Voisin et al. (2012) |
| Czechia | Mladeč | 52 | UPHS | M_3_ | L | 10.0 | Voisin et al. (2012) |
| Czechia | Pavlov | 1 | UPHS | M_3_ | L | 9.7 | Voisin et al. (2012) |
| Czechia | Předmostí | 3070 | UPHS | M_3_ | R | 10.0 | Voisin et al. (2012) |
| Czechia | Předmostí | 4 | UPHS | M_3_ | L | 9.9 | Voisin et al. (2012) |
| Czechia | Předmostí | 26 | UPHS | M_3_ | R | 10.0 | Voisin et al. (2012) |
| Czechia | Předmostí | 27 | UPHS | M_3_ | L | 11.14 | Voisin et al. (2012) |
| Czechia | Předmostí | 3 | UPHS | M_3_ | L | 11.9 | Voisin et al. (2012) |
| Czechia | Předmostí | 9 | UPHS | M_3_ | L | 11.8 | Voisin et al. (2012) |
| Czechia | Předmostí | 10 | UPHS | M_3_ | L | 10.2 | Voisin et al. (2012) |
| France | Abri Blanchard | no | UPHS | M_3_ | L | 9.30 | Voisin et al. (2012) |
| France | Abri Pataud | P1 | UPHS | M_3_ | L | 12.52 | Voisin et al. (2012) |
| France | Lachaud | 3.1980.6 | UPHS | M_3_ | L | 10.67 | Voisin et al. (2012) |
| France | Le Peyrat | Le Peyrat 5 | UPHS | M_3_ | L | 11.0 | Voisin et al. (2012) |
| France | Les Rois | R50.27 | UPHS | M_3_ | R | 10.1 | Voisin et al. (2012) |
| France | Les Rois | R50.3 | UPHS | M_3_ | L | 10.3 | Voisin et al. (2012) |
| France | Grotte de la Balauzière | L.B. XII | UPHS | M_3_ | L | 7.20 | Voisin et al. (2012) |
| France | St. Germain La Rivière | 4 | UPHS | M_3_ | L | 10.6 | Voisin et al. (2012) |
| Italy | Arene Candide | 5 | UPHS | M_3_ | L | 10.00 | Voisin et al. (2012) |
| Italy | Arene Candide | 2 | UPHS | M_3_ | L | 11 | Voisin et al. (2012) |
| Italy | Arene Candide | 4 | UPHS | M_3_ | L | 10.8 | Voisin et al. (2012) |
| Italy | Arene Candide | 20 | UPHS | M_3_ | L | 10.1 | Voisin et al. (2012) |
| Italy | Arene Candide | 19.6725 | UPHS | M_3_ | L | 10.2 | Voisin et al. (2012) |
| Italy | Barma Grande | 2 | UPHS | M_3_ | R | 10.5 | Voisin et al. (2012) |
| Italy | Barma Grande | 5 | UPHS | M_3_ | L | 10.6 | Voisin et al. (2012) |
| Italy | Barma Grande | 4 | UPHS | M_3_ | R | 8.8 | Voisin et al. (2012) |
| Italy | Grotte des Enfants | 4 | UPHS | M_3_ | L | 10.40 | Voisin et al. (2012) |
| Italy | Romanelli | R5 | UPHS | M_3_ | L | 10.70 | Voisin et al. (2012) |
| Italy | Romanelli | R7 | UPHS | M_3_ | R | 10.30 | Voisin et al. (2012) |
| Italy | Romito | 1 | UPHS | M_3_ | L | 10.60 | Voisin et al. (2012) |
| Italy | Romito | 5 | UPHS | M_3_ | L | 11.10 | Voisin et al. (2012) |
| Italy | Romito | 4 | UPHS | M_3_ | L | 10.00 | Voisin et al. (2012) |
| Italy | Romito | 3 | UPHS | M_3_ | L | 11.40 | Voisin et al. (2012) |
| Italy | Romito | 6 | UPHS | M_3_ | L | 10.20 | Voisin et al. (2012) |
| Italy | Romito | 2 | UPHS | M_3_ | L | 9.90 | Voisin et al. (2012) |
| Spain | El Mirón | El Mirón 1 | UPHS | M_3_ | L | 10.6 | Voisin et al. (2012) |
| Spain | Veyrier | 3 | UPHS | M_3_ | R | 10.28 | Voisin et al. (2012) |

**Table S4.** Crown and root diameters (in mm) of BH-5, BH-8, BH-7, and BH-15 compared with Neanderthal and modern human samples. Adjusted Z-score values above +1 or below −1, representing those outside the 95% confidence interval of the comparative group's variability, are highlighted in bold. For crown diameters, the comparative data are provided in **Tables S1–S3**. For root lengths, the data are sourced from Le Cabec et al. (2013, Table 4b) (original group names: “Neanderthals,” “Early modern humans,” “Upper Paleolithic and Epipaleolithic humans,” and “Recent modern humans”), except for early Neanderthal (i.e., Sima de los Huesos) I_2_s, which come from Lockey et al. (2023, Table 4). Abbreviations: BL = buccolingual crown diameter; LaL = labiolingual crown diameter; MD = mesiodistal crown diameter; *n* = number of specimens per sample; RL = root length; SD = standard deviation.

|  |  | ***n*** | **Mean** | **SD** | **Adj. Z-score** |
| --- | --- | --- | --- | --- | --- |
| **I^1^ RL** | BH-5 | 1 | 16.4 | – | – |
|  | Late Neanderthals | 17 | 17.23 | 2.4 | −0.16 |
|  | Upper Paleolithic and Epipaleolithic *Homo sapiens* | 6 | 13.50 | 2.09 | 0.50 |
|  | Recent *Homo sapiens* | 24 | 12.94 | 1.39 | 1.18 |
| **I^1^ MD** | BH-8 | 1 | 10.20 | – | – |
|  | Early Neanderthals | 20 | 9.55 | 0.50 | 0.61 |
|  | Late Neanderthals | 20 | 9.72 | 1.11 | 0.20 |
|  | Middle Paleolithic *Homo sapiens* | 3 | 9.77 | 1.16 | 0.08 |
|  | Upper Paleolithic *Homo sapiens* | 25 | 9.05 | 1.05 | 0.52 |
| **I^1^ LaL** | BH-8 | 1 | 8.80 | – | – |
|  | Early Neanderthals | 20 | 7.75 | 0.36 | **1.36** |
|  | Late Neanderthals | 20 | 8.71 | 0.62 | 0.07 |
|  | Middle Paleolithic *Homo sapiens* | 3 | 8.07 | 0.71 | 0.21 |
|  | Upper Paleolithic *Homo sapiens* | 25 | 7.60 | 0.40 | **1.43** |
| **I^1^ RL** | BH-8 | 1 | 17.90 | – | – |
|  | Late Neanderthals | 17 | 17.23 | 2.4 | 0.13 |
|  | Upper Paleolithic and Epipaleolithic *Homo sapiens* | 6 | 13.50 | 2.09 | 0.76 |
|  | Recent *Homo sapiens* | 24 | 12.94 | 1.39 | **1.69** |
| **I_2_ LaL** | BH-7 | 1 | 8.10 | – | – |
|  | Early Neanderthals | 20 | 7.29 | 0.42 | 0.90 |
|  | Late Neanderthals | 23 | 7.87 | 0.62 | 0.18 |
|  | Middle Paleolithic *Homo sapiens* | 4 | 7.25 | 0.47 | 0.51 |
|  | Upper Paleolithic *Homo sapiens* | 48 | 6.71 | 0.53 | **1.29** |
| **I_2_ RL** | BH-7 | 1 | 17.80 | – | – |
|  | Early Neanderthals | 16 | 17.53 | 1.10 | 0.11 |
|  | Late Neanderthals | 15 | 18.42 | 2.03 | −0.14 |
|  | Upper Paleolithic and Epipaleolithic *Homo sapiens* | 10 | 14.73 | 1.03 | **1.26** |
|  | Recent *Homo sapiens* | 47 | 14.09 | 1.42 | **1.28** |
| **m3 BL** | BH-15 | 1 | 10.20 | – | – |
|  | Early Neanderthals | 27 | 9.81 | 0.75 | 0.25 |
|  | Late Neanderthals | 38 | 10.98 | 0.77 | −0.49 |
|  | Middle Paleolithic *Homo sapiens* | 4 | 11.28 | 0.45 | −0.67 |
|  | Upper Paleolithic *Homo sapiens* | 39 | 10.41 | 0.87 | −0.12 |

**References for Supporting Information:**

Le Cabec, A., Gunz, P., Kupczik, K., Braga, J., & Hublin, J.-J. (2013). Anterior tooth root morphology and size in Neanderthals: taxonomic and functional implications. *Journal of Human Evolution*, *64*(3), 169–193. <https://doi.org/10.1016/j.jhevol.2012.08.011>

Lockey, A. L., Martín‐Francés, L., Arsuaga, J. L., Bermúdez de Castro, J. M., & Martinón‐Torres, M. (2023). Dental tissue proportions and linear dimensions of Sima de los Huesos lower incisors. *American Journal of Biological Anthropology*, *180*(3), 472–487. <https://doi.org/10.1002/ajpa.24651>

Martinón-Torres, M., Bermúdez de Castro, J. M., Gómez-Robles, A., Prado-Simón, L., & Arsuaga, J. L. (2012). Morphological description and comparison of the dental remains from Atapuerca-Sima de los Huesos site (Spain). *Journal of Human Evolution*, *62*(1), 7–58. <https://doi.org/10.1016/j.jhevol.2011.08.007>

Voisin, J.-L., Condemi, S., Wolpoff, M. H., & Frayer, D. W. (2012). A New Online Database (http://anthropologicaldata.free.fr) and a Short Reflection About the Productive Use of Compiling Internet Data. *PaleoAnthropology*, *2012*, 241–244. <http://dx.doi.org/10.4207/PA.2012.ART76>
